# African Female Physicians and Nurses in the Global Care Chain: Qualitative Explorations from Five Destination Countries

**DOI:** 10.1371/journal.pone.0129464

**Published:** 2015-06-12

**Authors:** Silvia Wojczewski, Stephen Pentz, Claire Blacklock, Kathryn Hoffmann, Wim Peersman, Oathokwa Nkomazana, Ruth Kutalek

**Affiliations:** 1 Department of General Practice and Family Medicine, Centre of Public Health, Medical University of Vienna, Kinderspitalgasse 15, 1090, Vienna, Austria; 2 Department of Family Medicine, University of the Witwatersrand, Johannesburg, South Africa; 3 Nuffield Department of Primary Care Health Sciences, University of Oxford, Radcliffe Observatory Quarter, Oxford, OX2 6GG, United Kingdom; 4 Department of Family Medicine and Primary Healthcare, Ghent University, University Hospital, 6 K3, De Pintelaan 185, 9000, Ghent, Belgium; 5 School of Medicine, University of Botswana, Botswana, Medical Education Partnership Initiative principal investigator, Gaborone, Botswana; Örebro University, SWEDEN

## Abstract

Migration of health professionals is an important policy issue for both source and destination countries around the world. The majority of migrant care workers in industrialized countries today are women. However, the dimension of mobility of highly skilled females from countries of the global south has been almost entirely neglected for many years. This paper explores the experiences of high-skilled female African migrant health-workers (MHW) utilising the framework of Global Care Chain (GCC) research. In the frame of the EU-project HURAPRIM (Human Resources for Primary Health Care in Africa), the research team conducted 88 semi-structured interviews with female and male African MHWs in five countries (Botswana, South Africa, Belgium, Austria, UK) from July 2011 until April 2012. For this paper we analysed the 34 interviews with female physicians and nurses using the qualitative framework analysis approach and the software atlas.ti. In terms of the effect of the migration on their career, almost all of the respondents experienced short-term, long-term or permanent inability to work as health-care professionals; few however also reported a positive career development post-migration. Discrimination based on a foreign nationality, race or gender was reported by many of our respondents, physicians and nurses alike, whether they worked in an African or a European country. Our study shows that in addition to the phenomenon of deskilling often reported in GCC research, many female MHW are unable to work according to their qualifications due to the fact that their diplomas are not recognized in the country of destination. Policy strategies are needed regarding integration of migrants in the labour market and working against discrimination based on race and gender.

## Introduction

International migration flows have increased considerably over the past four decades [1]. Between 1990 and 2013 the number of international migrants rose by 50% to 232 million [2]. The major destination countries are in Europe and Asia where nearly two thirds of the migrants live, followed by North America (53 million) and Africa (19 million). Almost 60% of the migrants who live in the northern hemisphere originate from countries from the southern hemisphere [2]. The factors driving migration are manifold and the concept of push and pull factors helps to understand processes of migration [3, 4]. Push factors emerge from the situation in the source country. They can be economic (poverty, unemployment, lack of basic health services), social and cultural (discrimination based on ethnicity, sexuality, gender), political (conflict, insecurity, violence, corruption) and environmental (natural disaster, harvest failures) [4]. Pull factors emerge from the destination countries; for example the falling birth rates in Europe account for an increase in demand and recruitment of migrant populations. The widening disparities in wealth on a global scale also play a major role [5]. Typical pull factors are possibilities of employment or a better standard of living [4, 6].

Emigration of health-workers has become an important global health issue. The loss of health-care professionals represents a threat for the health of millions of people especially in poorer countries [7, 8]. Wealthier industrialized countries fill care gaps with health personnel from other countries, often from the global south or poorer neighbouring countries. Recruitment of health-workers from the global south has increased in OECD (Organisation for Economic Co-operation and Development) countries since post-world war II [9]. That partly explains why the number of migrating women from the global south has also increased, as many work in care services [10–13]. Today, women represent half of the world’s transnational migrants [14]. Many of them are lead migrants (LM), especially in the care sector, which means they are the first member of the nuclear family to migrate, aiming to generate household income. Others migrate as accompanying spouses (AS), which means that they migrate for family reunification reasons where one partner gets a job abroad first and then the other partner follows. Female lead migrants from African countries like Nigeria and Ghana are also growing in number as entrepreneurial women or students [15–17]. As for the care sector, in 2007, about 90% of care-assistants in the US were women and a high percentage born in Africa or Central America [18]. In Europe, women made up more than three-quarters of the health workforce in 2010 with a growing percentage of migrants [19]. Moreover, women remain the principal informal care-givers to children, the elderly and the sick around the world [20, 21]. The migration of female healthcare workers thus results in significant losses in both professional and informal care of source countries [20–22].

The shortage of health workers in sub-Saharan Africa is especially severe. Around 65,000 African-born physicians and 70,000 nurses were working overseas in the year 2000 [23]. In addition, an OECD census in 2007 revealed that Africa has the highest percentage of migrating women with a tertiary education; highlighting that not only health-care workers are leaving but also highly educated women [22].

This paper explores the experiences of high-skilled female African migrant health-workers utilising the framework of Global Care Chain (GCC) research [24–29].

### The global care chain and female migration

Healthcare-worker migration represents the longest and best-documented female transnational labour migration [24, 27]. The organized export of female care-workers is documented as early as the 19^th^ century when the female workforce comprised nuns who travelled to countries of the global south as missionaries and nurses [24, 27]. However, the face of transnational healthcare worker migration has substantially changed in the 21^st^ century—female migration in the health-sector is now seen in the opposite direction, from the southern to the northern hemisphere and has been described by feminist scholars using the framework of the Global Care Chain (GCC). The Global Care Chain theory analyses international power relations between the global north and south that emanated from a changing transnational organisation of healthcare with a special focus on migrant women from the south and their incorporation in the global healthcare market [24, 27–31]. The term Global Care Chain was first used by the feminist sociologist Arlie Hochschild [32], referring to “*a series of links between people across the world based on the paid and unpaid work of caring*” (page 131).

The demand for migrant care-workers especially in wealthy industrialized countries is steadily increasing, largely due to an aging population and the failure of states to plan long-term and self-sufficient health-systems [18]. Also, the male-breadwinner stereotype has changed to a more gender equal model of dual-earner in some countries. Although the latter development is positive, it created an imbalance in roles: women have less time available for domestic work once they are part of the labour market, but this is not compensated by an increase in domestic contribution by men [33, 34]. As a result, the domestic care-work is handed over to others, mostly migrant women who work for families on a paid labour basis. Those migrant women then hand their own care responsibilities over to other persons, usually women from their families (elder daughters, mothers or grandmothers) or friends [35].

A lot of research on GCC has focused on migrant women from poorer countries working in private care settings [35, 36]. These female workers often face marginalization, difficult economic situation and unstable legal status in the host country [11, 18, 36]. Smith and Mackintosh [37] and Cuban [36] have exposed this trend in the UK.

However, the handing over of care tasks is not limited to private care. The global care chain exists in the public care sector as well. For a long time, public health-care in industrialized western countries depended on migrant care-workers because the national care workforce was not self-sufficient. Wright and colleagues [9] documented the extraction of care-work from South to North from as early as the 1960s on. Today, South-South migration has increased as well: many African nurses chose to go to upper middle-income countries like South Africa or Botswana [38]. Those countries are also highly dependent on a migrant care-workforce [23]. Also, the United Arab Emirates (UAE), a country whose economy is mostly driven by migrants and known to be recruiting health workers, are a major destination country for migrant nurses [38, 39]. There are studies from the UAE and the Asian Pacific Region that highlight the difficult situation for migrant women in that country, especially for domestic workers [40, 41]. The second field of GCC research, i.e. the public and institutionalized sector of healthcare (e.g. hospitals), focuses on the immigration of high-skilled healthcare workforce and how they fit into a global care economy. Only recently has research on care migration flow focusing on nurses increased [11, 24, 42–47]. A feature often reported by migrant nurses in highly institutional settings is the feeling of devaluation and relatively limited responsibilities compared to their home countries [29, 48]. Even in the more regulated environment of the institutionalized settings with labour rights, migrant nurses often worked in positions they were overqualified for [29]. Another important theme for GCC research is racial discrimination against black female migrant healthcare workers in their work environments [46, 49–51].

In the light of the abovementioned aspects our paper focuses on the migration of high-skilled female nurses and doctors, originating from sub-Saharan African countries, to European (UK, Belgium, Austria) or southern African countries (Botswana, South Africa). There have been few papers drawing on the experiences of foreign trained doctors from the global south migrating to middle- and high-income countries in recent years [52–56]. Female physicians have been entirely neglected in GCC research so far [24]. Moreover, research has mostly focused on Anglo-Saxon countries (as they are the major destination countries for migrating health workers), and barely on health-worker migration to other countries [24, 57]. The aim of our study was to explore the impact of migration on the careers of female migrant health-workers who had been trained in Africa. Furthermore, we wanted to compare the experiences of doctors and nurses and especially study their experiences of deskilling and discrimination.

## Materials and Methods

### Design

This study took place as part of a larger EU-funded research project on ‘Human Resources for Primary Healthcare in Africa’ (HURAPRIM) www.huraprim.ugent.be with seven partner countries. Semi-structured interviews with 88 female and male migrant health-workers were conducted in five out of seven project partner countries, namely Botswana, South Africa, the UK, Belgium and Austria between July 2011 and April 2012. In the overall study, the sample was heterogeneous according to gender, country of origin, age, training and time since migration to the destination country. For the purpose of this paper only the interviews with female doctors and female nurses were selected for further analysis (N = 34).

The interviews were conducted using a qualitative semi-structured guide that explored three main research questions: 1) personal experiences and reasons for migration of health-workers, 2) continued links with their countries of origin and 3) future (migration) plans. The interview guide was developed in English and translated into German, Dutch and French; interviews were conducted in the language preferred by the research participant (Dutch (n = 5), French (n = 8), or German (n = 6), English (n = 69)). The Belgian project team member who conducted the interviews also translated the interview guide into Dutch and French and WP checked the translation. The team member from Austria who conducted the interviews translated the guide into German and RK checked the translation. As the interview guide was semi-structured and aimed at framing the topics that needed to be mentioned during the face-to face interview the translation from experienced mother-tongue speakers was considered sufficient for the translation process.

Three of the countries where the interviews were conducted are among the top eight destination countries for migrant health-professionals (Belgium, UK, South Africa) [23]. Austria and Belgium have no history of active recruitment whereas South Africa, UK and Botswana have recruited healthcare workers in the past

### Recruitment of participants

The inclusion criteria for the European countries (UK, Belgium, Austria) were: 1) Born in sub-Saharan Africa 2) received professional training in medicine (physicians, nurses or midwifes) in sub-Saharan Africa (one interviewee was not born in sub-Saharan Africa, but had been trained in South Africa). The inclusion criteria for Botswana and South Africa were: 1) Born in another sub-Saharan African country 2) received professional training in medicine (physicians, nurses or midwifes) in another sub-Saharan African country. Participants did not necessarily have to be currently working in a health-related field. They could be working in another field, currently not be working at all, or be students. In the sample analysed for this study (N = 34), five respondents were currently not working in a health related field.

#### Austria and Belgium

The research teams in Austria and Belgium used locally adapted recruitment strategies to find potential interview-partners. In both countries a number of organizations were contacted: (African) migrant organizations, organizations offering counselling for newly arrived immigrants, language centres and organizations focusing on integration. Also, the teams asked nursing homes and hospitals to distribute flyers and identify potential participants. The call for participation was circulated in online fora and invitations were given out in neighbourhoods with high concentration of migrants. Also medical boards were approached like the Austrian Medical Chamber (ÖÄK) or the Austrian Nurses Association (ÖGKV). One of the more successful strategies turned out to be the snowball technique, where the interviewer asked for names and contact details of other potential study participants.

#### United Kingdom (UK)

Recruitment was approached using two phases. In phase one adverts and posters were displayed and emailed to targeted groups and individuals. National professional and support groups, local health service employers, and appropriate individuals known to the research team were contacted. Snowballing was used wherever possible. In phase two, individuals expressing an interest were sent a study information pack including information about the study, a response form and a consent form.

#### Botswana

Letters were sent to the Ministry of Health, District Health Management Teams and District Council Secretaries as well as to the nursing and midwifery council of Botswana to explain the study and request permission to recruit health workers for the study. Notices were put on the institutions inviting volunteers. Those who were interviewed were asked to recommend other health professionals that could be approached by the study team.

#### South Africa

The research team from the University of the Witwatersrand in South Africa started with a list of registrars at the Department of Family Medicine as that is one of the few specialties that foreign doctors are able to register for. The District Family Physician in each of the five districts of Gauteng Province was contacted for an additional list of names. Human Resource Managers in major hospitals in Johannesburg were also contacted. Once again, snowball sampling proved to be the most effective means of sampling for participants.

### Data collection

In all countries researchers with experience in qualitative research conducted the interviews. The researchers involved in the interviews (conducting, transcribing and analysing) were post-graduate anthropologists and sociologists well-trained in qualitative research methods. Two of three interviewers in the UK for example had attended a qualitative research course (MSc level). Participants were interviewed for 60–90 minutes in English, Dutch, French, or German in a place of their choice, usually work or home. Interviews were recorded and transcribed verbatim by the interviewers themselves or by other team members. Some of the participants in Botswana were telephone-interviewed as it was not possible for the team to arrange a face to face interview because of the distance of their villages from Maun, where the team was based.

### Data analysis

The 34 interviews analysed for this paper were imported into the software atlas.ti for qualitative content analysis. One researcher (SW) analysed the interviews and coded them into themes and subthemes [58, 59]. While reading through the interviews a total of 63 codes were defined. For the purpose of this study 27 codes were summarized and analysed under the three overlapping categories “migration and impact on women’s career”, “gendered work dynamics pre and post migration” as well as “experiences of racial discrimination” (see S1 Table). Most of the codes were defined deductively according to themes that emerged from the Global Care Chain literature focusing on high-skilled female health migration (e.g. abroad_racism or abroad_impact professional) and some codes emerged from the interviews using an open inductive coding approach (e.g. abroad_difficulties, personal details). To make the coding scheme and theme development valid and reliable, it was discussed with another researcher familiar with the data material and with extensive knowledge in qualitative methods (RK). The codes were merged into categories and summarized by SW [60]. After several iterations with the co-authors who conducted or were familiar with the content of the interviews the results were finalised.

The results presented below include many original texts/excerpts from the interviews (see also S1, S2, S3 and S4 Texts for original quotes). By doing so, we aim to give the interview partners in the research as strong a voice as possible [61].

### Ethics statement

Ethical approval was provided by the ethics committees of each of partners mentioned in the study design section. We here specify in detail ethics committees/approvals related to the five countries included in the study for the paper: Ghent University, Belgium (Ref.: 2011/552), University of Oxford, United Kingdom (MSD/IDREC/C1/2011/96), University of Botswana (PPME 13/18/1 VII (368)), Medical University of Vienna, Austria (EK-Nr: 989/2011), and the University of the Witwatersrand, South Africa (M111122)). The data produced in the project is strictly confidential, and interviewees are anonymous in all transcripts and analyses. To assure anonymity/privacy, names that appear in the results section are all fictitious and we made transparent only the destination country of our respondents. Prior to the interviews, all participants were informed about the HURAPRIM project and its objectives as well as the purpose of the interviews. Written informed consent was obtained from all participants.

The participants were informed about the use of the data for scientific publications.

## Results

Thirty-four interviews with female migrant nurses and physicians from sub-Saharan Africa were analysed. Participants were between 26 and 70 years old; most were between 30 and 50 years. This age group had particularly significant family responsibilities, with many having children. Participants had lived in their country of destination for between one and 34 years, with the majority living there for more than ten years (reference year 2012) (see Table 1).

**Table 1:** Sample characteristics.

**Age**	
26–30	1
31–40	14
41–50	10
51–60	7
61–70	1
70+	1
**Time since left country of origin**	
1–4 years	5
5–10 years	12
11–20 years	14
21–30 years	1
31–34 years	2
**Country of origin**	
Angola	1
Congo	1
DR Congo	7
Gabon	1
Guinea	1
Ivory Coast	1
Malawi	1
Rwanda	1
South Africa	9
Sudan	1
Tanzania	1
Uganda	1
Zambia	6
Zimbabwe	2
**Country of destination**	
Austrias	6
Belgium	4
Botswana	6
UK	9
South Africa	9
**Medical profession**	
Physician	13
Nurse (Midwife)	21

As the interview sample was recruited purposively the numbers differ from country to country. There were more interviews with female health workers in the UK and South Africa as they are in general major destination countries for migrant health workers. Hence, interview partners were easier to find than in atypical destination countries such as Austria. Regarding the countries of destination the countries of origin South Africa, Zambia and Democratic Republic (DR) Congo outnumber other countries by far. This is the result of special bilateral agreements between these three countries and some of the countries of destination like South Africa, Belgium and the UK. Also, nurses have been migrating for a longer period of time already and migrant nurses outnumber migrant doctors in general, therefore we were able to recruit more female nurses than female physicians [23].

There were more female lead migrants (LM) in the sample (n = 21) than accompanying spouses (AS) (n = 13). The proportion of lead migrants within the group of nurses was particularly high; almost 2/3 of the nurses, and more than 50% of the migrating physicians were lead migrants in our sample (see Table 2).

**Table 2:** Migration status.

Respondents	Lead migrant (LM)	Accompanyingspouse (AS)	Total
**Total**	**21**	**13**	**34**
Physicians	7	6	13
Nurses	14	7	21

### Migration and impact on women’s careers

#### Temporary inability to work, deskilling & devaluation

Many of our respondents experienced at least temporary deskilling or inability to work due to a long recognition process of their qualifications, waiting two to ten years before being able to practice as a physician or nurse. A case of temporary deskilling is reported by a physician in the United Kingdom:

When I got to the UK, the references are difficult to get in the beginning; even getting jobs is very difficult because in the beginning I had to do care work for, I don't know, for some long time, about four months or something. I did care work.

(Grace, physician, UK)

For Helen, a physician living in Austria, it took ten years to restart her career and now she still has to do three training years in Austria, although she already completed them in her home country. When Helen talked about her life and career, she mentioned what Meares [49] (page 473) calls a “re-domestication”:

It’s difficult, because I was so active in my country, and here, I found myself, without work, practically being a housewife. Suddenly I was a housewife. And also, having kind of a bad conscience, I am a physician, and I can’t do anything. Knowing that my colleagues are all continuing to work, for me it took very long, I was in Austria for one and a half years, then I got my second child in 2001, and then it was for me, almost the depression [laughs].

(Helen, physician, Austria)

Post-migration, Helen’s professional and personal situation changed considerably. She first had to learn a new language and could only then undertake the whole recognition of diploma process. Combined with private circumstances, like having and taking care of children and the household while her husband was earning the income for the family, it took ten years to restart her career. She still has to do three years of training in Austria, although she had already completed them in her home country. Helen enjoys being a mother; however, she always wanted to pursue her career as well. The fact that she had to stay at home because of these circumstances was very frustrating for her. Despite the long process she decided to make the effort in order to work as a physician, which is an indication of how important her work is for her identity.

Temporary deskilling or inability to work also affected nurses in our study. In South Africa most of the interviewed nurses had to wait at least two years before being allowed to work. The foreign certificates have to be handed to SAQA (South African Qualifications Authority) and then the equivalence is made with South African standards. That process can already take almost one year. Then the nurses have to register with the nursing council. Some respondents waited up to one year to get an answer from registration. What Linda reports in the quote below was experienced by many other nurses but also physicians in our study who wanted to work in South Africa. Whether they passed the qualification test quickly after their arrival or not, most of them waited at least two years before being able to apply for jobs in South Africa.

To get feedback from the nursing council it is hell, it was not easy until we had to travel to and from, to and from and it was costly and also like mentally we were disturbed because we could not do our clinical practice because we needed that registration process first.

(Linda, nurse, South Africa)

I came here to register as a nurse. It took me 2 years because it’s not all that easy. They really have to prove that you are a nurse where you come from.

(Susan, nurse, Botswana)

Some of the participants also experienced a devaluation of their job; this was reported by nurses in the United Kingdom especially.

They can't put a nasogastric tube and it's a trained nurse. In South Africa your first year or you know, you can put a nasogastric tube; you know those kinds of things. So we've come here with all that knowledge, […], I was showing them that I can do this and then when you come here, no that's it, you cannot even give Paracetamol.

(Irene, nurse, UK)

And I think partly being a training hospital, I think I've lost a lot of my skills because I used to do an awful lot more in intensive care than you'd ever be allowed to do here or even in the emergency department really.

(Betty, nurse, UK)

There are a number of studies that support Betty’s and Irene’s view. Part of the reason of feeling devalued might be the fact that many of the African nurses cannot use all the skills that they acquired due to different rules in European health systems where there seem to be more restrictions regarding who is allowed to perform certain medical tasks [37, 46, 62]. Another reason can be that nurses with an African diploma are often placed in jobs which they are overqualified for. The loss of professional and social status is reported for example in the study from Aboderin [62] for Nigerian nurses working in the UK. The interviewed nurses expressed the feeling that nursing was highly valued in Nigeria and felt that this was not the case in the UK. O'Brien [46] reported the frustration of nurses from the Philippines and India that were not able to use the whole spectrum of their skills (e.g. IV) in the UK NHS services.

#### Permanent change of career path

Four respondents had been totally unable to work as physicians due to bureaucratic challenges for Non-EU citizens. They were still awaiting recognition of their qualifications and had decided to pursue a different professional path instead—a job where they would be deskilled.

Yolanda is a physician and came to Belgium as a single, lead migrant more than five years ago due to personal health problems and better opportunities of treatment. Her experience shows a lose-lose situation for both the migrant and the destination country in Belgium.

They don’t accept doctors with a non-European diploma. I understood that quite fast that it was not possible. So I looked at possibilities to re-take my studies here. I really wanted to work. So I did some research and found out that I would have to re-take four years of studies. There is an exam where they only take 15 persons for the whole of Belgium. I thought, ‘That I can do!’, but four additional years of studies, I did not agree. I had a good education—4 years, I really think that is too much.

(Yolanda, physician, Belgium)

Another example is the physician Sonia who came to Austria more than five years ago. Although she had many years of experience as a physician, she still has to repeat a lot of classes before her diploma will be recognized and she has to pass a study test in medicine in german. This process of diploma recognition is called ‘Nostrifizierung’ in Austria. After passing that, the Austrian government can decide if the applicant has to study even more or if the applicant can start the three years of training in hospital (called ‘Turnus’) as a medical doctor in Austria.

Here no, I'm not allowed to practice or to work as a doctor because I have to do the “nostrification”, it is a long process […]. So I can't do it right now. I can't work, I'm not allowed to work as a doctor.

(Sonia, physician, Austria)

For nurses, the permanent inability to find a job according to their qualifications was rare; most of them found one after a shorter or longer waiting time. However, there were exceptions. Angela, a nurse, came to Europe to complete a post-graduate training in Public Health more than 30 years ago. She had already worked in her home country in many health-care settings as a nurse. When her husband completed his studies in Belgium they went to an African country where she was easily able to work as a nurse in maternity. After ten years, the family with two children went back to Belgium for health reasons of their daughter and because they wanted to give their children a good education. Today, Angela’s children and grand-children live in Belgium. Angela wanted to work as a nurse when the family returned to Belgium, but her diploma was not recognized there.

Yes, that is the problem. I tried to get information, to do the equivalence but the answer was always ‘You have to repeat one year of education’ or ‘You have to repeat all the practical trainings’. Everything I already did, I would have had to re-do it. So I said: ‘That’s not possible!’ That is when I decided to take the job as a care-assistant.”

(Angela, nurse, Belgium)

Angela considered the barriers to fulfil the Belgian requirements as too high and decided to take a path where she would be professionally deskilled. To be able to work as a nurse in a hospital setting she would have to re-do the practical trainings or one year of the education cycle. After so many years of experience she considered that was too much to ask of her. Besides, Angela had considerable private care responsibilities with her children (and now also grand-children) and the perspective of having to study again at her age (between 50 and 60) after so many years of practicing was not attractive to her. In addition, Angela had alternative professional plans and reinvented herself: she founded an organization for migrants.

There is the case of Sophie (between 30 and 40), who came to Belgium as a formal political refugee more than ten years ago, when she was almost finished with her training as a nurse. The requirements to work in Belgium were to complete three years of training as a nurse. This requirement however was not easily to fulfil as she had to take care of her children as well. She came to Europe alone as a lead migrant with two children. So there were the private obligations of care and the fact that three years of additional training were a long time considering her precarious economic situation. Weighing her options, she decided that when her children were be a bit older Sophie would take up the training as care assistant, which will take two years in total with one year at an institution where she would already get paid.

The problems of deskilling or inability to work for professionals with non-European diplomas have been discussed in many studies [29, 36, 37, 48, 50, 52, 63]. Ouali [64] argues that particularly migrant women with a non-European graduate diploma have a hard time finding a job as the European market is overcrowded with highly qualified personnel, and the EU favours European diplomas over non-European ones. The professional career developments are often jeopardized for non-European women post-migration and they often take jobs below their qualification profile [36, 64]. What is rarely reported in other studies is that professional African women migrating to other African countries (South-South migration) can also be affected by inability to work or deskilling, as shown by our examples Linda and Susan (South Africa, Botswana). There is one study from the ACMS (African Centre for Migration Studies) that states that international female migrants in South Africa (who often come from other African countries) are on the one hand often well-educated; on the other hand they often face the risk of underemployment and discrimination on the labour market [65].

#### Fast career development post-migration

The processes of deskilling or the (temporary) inability to work in the qualified health profession post-migration is by no means universal; in a few cases, the experience of migration allowed women to improve their careers (more so for nurses than for physicians).

It was actually easier to come to South Africa soon after the nineties than it is now. Because actually at the time, you know it was very simple, the South African Medical Council offered an exam, you wrote, if you passed the exam then you were eligible to register and you could apply for a job and assuming you were given a job offer, home affairs will then, you know, give you a work permit.

(Beth, physician, South Africa)

What Beth is describing is a feature shared by some other respondents too, also in European countries. When the respondents migrated some 15 years ago it was seemingly easier to get their diplomas recognized. One explanation is that in that time there were no international political requirements to restrict migration of health workers from sub-Saharan African countries that face health worker shortages; those requirements resulted in the restriction of recruitment in the UK or South Africa only recently.

Other experiences of positive career development were reported in Austria:

In fact I almost always worked in managing positions with only brief interruptions; as deputy head nurse or head nurse […] and now here also in a leading position.

(Catherine, nurse, Austria)

It worked out well in nursing school [in Austria] […] I knew that I would finish sometime soon and I always had a good feeling, because the conditions in our country are tough and we have to pay for the education. Here in Austria I did not have to pay. […] For my first job I was surprised in a positive way: I applied for jobs everywhere and I was accepted almost everywhere as well.

(Rachel, nurse, Austria)

Both examples are somehow special: Catherine had already worked 17 years in another European country as a hospital nurse and because of that fact it was easy for her to find a job in an adequate position in Austria. As for Rachel, she had to re-do most parts of her training as a nurse. She was very young when she migrated to Austria for political reasons. The efforts to re-do the training were acceptable as she was still at the beginning of her career when she migrated and without private care responsibilities for children. Also the feeling of gratitude towards the country of destination that “accepted” her and not wanting to jeopardize the living in the host country, a country where life for black Africans is already characterized by a lot of discrimination, might also play a role in the narration of Rachel [66, 67].

### Gendered work dynamics pre- and post-migration

Gender themes were sometimes discussed during the interviews without specifically being asked. The stories show a very diverse picture of gendered dynamics (at work) pre- and post-migration:

The decision to leave X was actually two because it was a time, I had just divorced and you know, heartbroken and those years when you were divorced you were regarded as like a low person, low class; a married woman didn't want to associate with you, single women didn't want to associate with you; there was a name that was given to us, a very derogatory name in our language,[…]. and then you just wanted to break away and go away and hide so that these people don't know you are divorced. And then this country was recruiting.

(Irene, nurse, UK)

Irene went to the UK with a recruitment agency, which represents a pull-factor; however, it is important to note that gender stigmatization remained a very important push-factor. In her position as a single, divorced mother of three children she felt isolated and stigmatized in the public as well as the private sphere. Professional career development in her country of origin did not seem to be an option at the moment of migration due to that stigmatization. Only because of the push factors, the pull factors came into play.

In other cases, gender mattered post-migration, particularly in regards to the differences in culture experienced:

At times I find it very difficult to express myself so there's some challenges at work really. The socialising culture is a bit different here than in X. Things like going to the pub, a part of life here; so you have to end up trying to fit into the group and do some of the things which you wouldn't normally have done in X. A pub culture is not [for a] lady, it's there for men, men go to the bar everyday but women don't, it's really different. But I've got used to it now. When I tell my friends in X they can't believe what I'm telling them.

(Grace, physician, UK)

It is really very hard to live in X [first country in Africa she migrated to] as a woman. And I was with my mother and it was hard also to find a job, because they manipulate everything. As a woman without [a] man to live in X is very hard, there is no respect, the community is very complicated.

(Sonia, physician, Austria)

The problems that Sonia mentioned were only raised by her. However, she did mention that feeling of being discriminated as a woman in her job in a former non-European destination country many times during the interview, explaining that it is tough to work as a female (and single) physician surrounded by mostly men. That view is reflected by other studies for example concerning gender-based discrimination of female physicians relating to career advancement possibilities in the US or Japan [68] and in general exclusion of female physicians from power structures of medicine [69] or sexual harassment against women working in the medical field [70].

The point raised by Grace, the physician in the UK, was unique and highlights the different gender cultures one finds when migrating and how to adapt to a new gender culture. Cultural and social constructions of womanhood or manhood differ across countries and regions, therefore a migration also includes being introduced to a new gender culture and having to renegotiate personal gender identities [71, 72].

### Experiences of racial discrimination

Many of our respondents reported experiencing discrimination. Some respondents voluntarily shared their experience of not feeling welcome as a foreigner, whereas in other cases this aspect was deduced from the stories themselves. Here are some examples from female doctors:

Some of those issues you have, you can't talk about them in the team, nobody, some people wouldn't know what you're going through as a foreign person working in this country because at times I hear my colleagues say, ‘Why do these foreign doctors come?’ […]

(Grace, physician, UK)

To put this statement into a larger context, it is important to note that respondents with a white European heritage did not mention experiences of discrimination based on being a foreigner which indicates the nature of what Grace talks about—it is not only about being a foreigner, it is about being black. During the interview Grace talked about one particular person that was harassing her; she did not mention any other person correcting this harasser or defending Grace’s case. Racial discrimination against black Africans is also mentioned by respondents in South Africa and Botswana. Beth thought that the university was very welcoming but other parts of social life were not easy for foreign Africans in South Africa. Rita, a physician in Botswana, when asked about what she would change in Botswana, mentioned the behaviour of both co-workers and patients.

From a social sort of environment point of view I think there are issues about—I don’t know about how other Africans from the rest of Africa experience it—but I know my experience resonates with many of my fellow Zambians, Zimbabweans and Kenyans. It’s an ongoing struggle and one just, you sort of get tired of it and think it’s time to go back home. (Beth, physician, South Africa)

The thing is just if they can teach the patients to respect us doctors; doctors and nurses. We know we are foreigners, but we are giving good service here if they could just respect us I will be happy to work here in Botswana, even to stay here forever.

(Rita, physician, Botswana)

Nurses had similar experiences and feelings of being discriminated against. The different narrations show a variety of features of racial discrimination. The narration from Irene illustrates how racist behaviour is to be found in the public sphere in general as well as in the work place. No environment is safe from it. It can come as an attitude from co-workers (see quote Marian), consultants (see quote Catherine) or from a random person at the airport (Irene).

By the time I left X I was a senior nurse so you come to a new country whereby, first of all, people really treated you as an underdog. I don't like to say but it was the effect that because of your colour; because you came from Africa, the third world. Somebody would ask me, ‘Oh where did you buy your clothes, did you buy them at the airport? Do you wear clothes in X; I thought you wear, you know, those kinds of things. (Irene, nurse, UK)

Hatred between co-worker and co-worker more especially when it comes to foreigners. Foreigners, we are not loved in Botswana. […] you find someone is asking ‘Ah! You! […] you are eating our money!’

(Marian, nurse, Botswana)

And the consultant back then, I remember the words she used were so offensive: ‘You do not have to repeat nutrition classes, because after all you lived in [another European country] for 17 years.’ As if we in X wouldn’t know what vitamins are or what nutrition means. Those are people that simply have no idea, they only look at the paper […]. Really, a horse can see more; those people wear those blinkers and don’t see what is going on beyond them.

(Catherine, nurse, Austria)

A similar experience at the workplace shared by Uma was reported in a study from Aboderin [62]. The author mentions interview reports from struggles over authority between white carers and black Nigerian nurses in the UK.

Working in the ward with somebody who'll tell you that, ‘Oh you are a registered nurse? No but I'd been a carer in this ward for more than ten years so don’t come with an attitude of thinking you're going to run this ward. I'm in charge here.’ There's an exchange of roles because I'm a nurse and I qualified in X, together with the fact that I'm black, I cannot work as a registered nurse with the healthcare-assistant who's been there for ten years. […] “

(Uma, nurse, UK)

Racial discrimination makes the struggle for integration, creating a positive social environment, finding a job and feeling at home even more difficult than it already is for transnational migrants, as the account of Beth shows: “it is an on-going struggle and you get tired of it” (Beth, physician, South Africa).

A major problem in all the stories seems to be that there is no strong public condemnation of racial discrimination at the work place. An anti-racist consensus in the work places is obviously missing. However, Uma underlines in her interview that the situation was worse when she arrived many years ago in the UK where she had no institutional or political support to condemn racism at the work place, which indicates that some change happens at least at institutional level.

## Discussion

This is the first study that jointly explores the migration experiences of African female physicians and nurses using a qualitative approach, featuring the phenomenon of female high-skilled international migration in the health-sector. In the context of the Global Care Chain, our results show that besides processes of deskilling and devaluing of female care-workers from the global south [24, 27–31], the biggest challenge for our respondents was temporary or permanent inability to work due to lack of diploma recognition in the destination country. This was particularly true for migrant physicians. Research about career development of skilled migrants suggests that migration tends to have a worst effect on the women’s than on the men’s careers [49]. However there are recent studies that indicate that in the health-care sector the impact on the career tends to be comparable in many regards between men and women [52, 57]. One thing particular to migrant women though seems to be the phenomenon of re-domestication post-migration [73].

Deskilling and devaluing of overseas nurses has been reported in various studies [25, 29, 46, 62, 74]. Studies about these phenomena for migrant female physicians, however, are missing. In some cases, our participants were permanently unable to find a job according to their qualifications from their home country; they were often faced with the decision to either take a menial job or wait years for their qualifications to be recognized. The humiliation of not being recognized as a physician or nurse was so strong in some cases that participants reported giving up on the option of working as a health professional in their destination country. In general, it was easier for the nurses to find a job according to their qualifications than for physicians. One reason for the difficulty in finding jobs could be that regulations and qualification requirements for health professionals are highly regulated at national level and these may be more stringent for physicians than for nurses. Also, policies are motivated by specific domestic interests: In the European Union the demand for nurses (and other care personnel) is larger than the demand for physicians and therefore the recognition process may be easier for nurses [19].

Some of the countries also have a long history of recruiting overseas nurses; in fact there is a common nursing curriculum within the Commonwealth (which includes 18 African countries), according to Wright and colleagues [9]. In general, skilled health-worker migration illustrates very well how colonial history shapes the migratory processes of today, as health-workers in many cases migrate to former colonizer countries [10, 13, 24, 27, 28, 53].

There are several studies that highlight permanent inability to practice as a physician with African diploma in OECD countries [75, 76].

In countries like Austria or Belgium, recognition could take two to three times longer than in countries like the UK [75]. In Belgium an additional course is necessary after taking an exam [77]. From 2001–2006 there were, for example, 16 applications of physicians originating from DR Congo, and only one was considered to have the equivalent of a Belgian diploma [77]. Although the process might be faster for nurses than for physicians, it is far from easy being recognized as a nurse in Belgium. For Austria the criteria for the recognition of qualifications (nostrification) are the content, range and formal requirements of the Austrian medical degree (ENIC NARIC AUSTRIA 2012). Without the nostrification and German language skills, health-workers are not allowed to work in Austria. Citizens from the EU get their diplomas automatically recognized, as for them the directive 2005/36/EG of the European parliament and council from 2005 applies.

The South African Department of Health released a new policy in 2006 regarding the employment of foreign health professionals in the South African health sector [78]. It attempts to restrict the recruitment of foreign health workforce from developing countries without specific government agreements and aims at mitigating negative effects from brain drain in developing countries (this policy was renewed in 2010 with further restrictions). This policy adversely affects job opportunities for migrant health workers and favours some migrants over others, as the policy restricts recruitment unless there are specific bilateral agreements [78]. It seems that the South African policy focuses on the restrictive measures regarding brain drain and leaves out the measures focusing on cooperation and strengthening of regional partnerships. The Health Professions Council of South Africa (HPCSA) also particularly states that it does not encourage recruitment of foreign health professionals from developing countries as anti- brain drain measure; however the reality is that the majority of the persons wanting to register are from those countries most restricted by the policy [79]. Many doctors from Botswana for many years have been trained in other countries sponsored by the Botswanan government but the challenge is that most do not return to Botswana upon graduation [80]. The country actively recruits doctors from many foreign states (mostly in sub-Saharan Africa) but they still have to pass the registration examination. In 2000, 70% of physicians in Botswana’s public health-sector were foreigners [81]. Botswana however has trained nurses locally for many years and more than 80% of the nurses in the country are from this country [80].

Some of our respondents reported experiences of discrimination based on gender in both source and destination countries. The phenomenon of the gendered division of labour in industrialized societies where men are responsible for the public space and women are responsible for the private space is still prevalent in many ways, no matter how much women participate in the labour market [34]. The experiences of our respondents are a reflection of power relations based on gender. The physician sector in many countries is still male dominated and women are often marginalized when they decide to go into a profession like medicine [68, 70]. It is however noteworthy that there are positive developments and that in some countries there is a gender balance in medical schools and some specialties (e.g. U.S., South Africa) [82].

Although gender mattered as a category of marginalization in the experiences of our interview partners, race was by far the most outstanding category of oppression reported by the female respondents when talking about challenges in the destination countries. White African migrants did not report discriminations based on gender or race in the destination country (although the non-reporting does not mean that they did not actually have experiences of discrimination). That result is supported by findings from Fauvelle-Aymar [65] who states that gender discrimination affected mainly the black African communities in South Africa. Economic marginalization was hardly ever mentioned by our respondents, which shows that they belong to a group of migrants that are better off (physicians more than nurses). Still it was mentioned in some cases as their working situation was or is precarious due to the long periods of diploma recognition and short-term contracts.

Hence, while our respondents seemed to be rather privileged in some aspects (class) other aspects of marginalization were much harder to cope with (race and gender) and had negative effects on private, emotional and professional lives [83, 84]. Racial discrimination was one of the reasons why respondents were wishing to return to their countries of origin in some cases. All our black female respondents had experiences with racial discrimination and coping strategies of handling the feeling of being viewed as inferior or as an outsider. It is very important to listen to these narratives of everyday discrimination to understand the magnitude and the multitude of faces of discrimination against black African women and to raise awareness of their often unfair treatment [83–86]. Working with personal stories of migrants helps to illustrate shared difficulties that migrant groups face globally but also shows that every story happens to an individual with a different background [37, 46, 50, 51, 62].

A limitation of the study was that participation of our respondents was voluntary leading to the possibilities for selection bias. However the qualitative sample offered rich information and showed a variety of circumstances. The data is based on self-reports by participants, thus being subject to possible recall bias; the findings therefore are not generalizable. However, in connection with the literature presented the findings show some tendencies that seem to account for a reality lived by many migrant health workers from sub-Sahara Africa working in high or middle-income countries.

In conclusion, our results suggest that on the one hand many African countries lose female care workforce and on the other hand European and other African destination countries do not properly include those migrants in their labour markets and civil societies. The migration of high-skilled care workers thus often becomes a multiple loss situation.

Policy strategies are needed regarding integration of migrants in the labour market and working towards anti-discrimination based on race, gender and other categories. Policy strategies regarding anti-discrimination have to be grounded in the similarity and the distinctiveness of inequalities [87]. That means, for example, that the social categories race, class and gender need to be framed in distinct policy strategies. For our interview partners that would mean to improve efforts of legal actions against racial discrimination at the work place and civil society in general and to improve mechanisms of diploma recognition for non-OECD country citizens to decrease global power imbalances. The majority of our interviewed female health-professionals managed to find jobs in the destination country; but only with a long recognition of qualification and/or deskilling process involved, no matter how high their level of education or comprehensiveness of work-experience. Some good practices in addressing the needs of migrant women were shown in a United Nations Population Fund [20] report. Two of the recommendations of the report seem very suitable for our paper: 1) Promote better knowledge, perception and self-representation of migrant communities, in order to foster more consistent integration between migrants and civil society; 2) Improve social and work-related integration of migrants and enhance the quality and access of public services for migrants through sensitization and training (page 121).

Future research regarding health worker migration should focus more on the personal circumstances of women and men, how they combine job and private care responsibilities in the destination country; the conditions of care arrangements and networks back home and how they changed with the migration and what support they would need in the destination country.

## Supporting Information

S1 Table Categories with codes. (DOCX)

S1 Text Quotes doctors_career. (RTF)

S2 Text Quotes nurses_career. (RTF)

S3 Text Quotes doctors_difficulties. (RTF)

S4 Text Quotes nurses_difficulties. (RTF)
