# Supplementary material for: African Female Physicians and Nurses in the Global Care Chain: Qualitative Explorations from Five Destination Countries
Source: PLoS One. 2015 Jun 12;10(6):e0129464. doi: 10.1371/journal.pone.0129464 (PMC4466329; doi:10.1371/journal.pone.0129464)
Supplement: S1 Table — (DOCX) [file pone.0129464.s001.docx]

**Supporting information**

**S1 Table.**

**Category with codes: names and descriptions**

**S1 Codes: names and descriptions**

| **Name of Category with codes** | **Category/code description (several codes could be attributed to the same quote)** |
| --- | --- |
| **Migration and impact on women’s career** | **How was the career development of the women post-migration?** |
| abroad_difficulties_prof_emotional | Post migration: Encountered difficulties, professional and emotional |
| abroad_division of work | Post migration: Division of work public/private, gendered |
| abroad_family constellation | Post migration: family constellation |
| abroad_imp_professional | Post migration: professional development abroad, professional impact of the migration |
| abroad_living con | Post migration: living conditions |
| abroad_private life | Post migration: organization of private life |
| abroad_resident status | Post migration: resident status |
| abroad_training_pos | Post migration: training, positive stories |
| abroad_training_neg | Post migration: training, negative stories |
| abroad_work con_neg | Post migration: work conditions |
| abroad_ work exp general | Post migration: work expectations |
| home_division of work | Pre migration: the division of work public/private, gendered |
| home_support_private | Pre migration: private support networks |
| personal details | Personal details of participant (age, number of children, profession, etc.) |
| reason_migr_political | Reason for migration: political |
| reason_migr_private | Reason for migration: private (e.g. love) |
| reason_migr_pull | Reason for migration: pull factor |
| reason_migr_push_econ | Reason for migration: push factor economic |
| reason_migration_studies | Reason for migration: study purpose |
| reasons_migr_prof | Reason for migration: professional |
| **Gendered work dynamics pre and post migration** | **Did and if so how did gender play a role pre and post migration privately and professionally** |
| abroad_gender | Post migration: narratives were gender played a role (work or private environment, division of labour) |
| hc_gender | Narratives were gender mattered at work in health care in the home countries |
| home_gender | Pre migration: Narratives were gender played a role (work or private environment, division of labour) |
| home_decision to become a doctor/nurse | What influenced the decision to become a doctor/nurse? |
| **Experiences of racial discrimination** | **Do the respondents tell personal stories about discrimination? If so how and what are these stories like?** |
| abroad_racism | Post migration: narratives of racism against the respondent |
| home_racism | Pre migration: narratives of racism against the respondent |
| abroad_inequalities | Narratives of inequalities that the respondents felt they had experienced |
| abroad_difficulties_general_prof_emotional | Post migration: Encountered difficulties, professional and emotional |
