# Supplementary material for: African Female Physicians and Nurses in the Global Care Chain: Qualitative Explorations from Five Destination Countries
Source: PLoS One. 2015 Jun 12;10(6):e0129464. doi: 10.1371/journal.pone.0129464 (PMC4466329; doi:10.1371/journal.pone.0129464)
Supplement: S1 Text — (RTF) [file pone.0129464.s002.rtf]

Original quotes from female physicians - career


P 2

Ja, ich war müde, aber das hab ich nicht gespürt, und es war, nicht nur für mich, eine Bereicherung, sondern für meinen Sohn auch. Der war 20 Monate alt als wir [lacht herzlich], dort gegangen sind.  20 Monaten war er und, ein Jahr später sind wir zurück, nach Wien, weil einfach weil, ich hatte die Chirurgie vermisst. Ich wollte mehr operieren und so ein Jahr lang hab ich fast nicht operiert und deswegen wollte ich meinen Vertrag nicht verlängern lassen. 00:12:58-5 


P 2: 
un, weil, ich fühle mich wohl hier und so, und nur, ich wollte mehr, weitermachen, ja, und es war auch/ der Junge hat das nicht gespürt, mein Sohn war gerade eins, ein Jahr und acht Monaten als wir zurückgegangen. Er hatte Papa einmal gesehen, ja, aber irgendwie fehlte ihm den Papa. Mein Mann konnte nicht öfters runter, runter fliegen. Aber i denk mir, mehr, wiel ich einfach mehr operieren wollte, ja. 


P 4

P4: Schwierig. Schwierig. So, ich könnte keine deutsch sprechen, und so, und dann so ich hab im x hab ich meine Tochter bekommen, und ja es war für mir schwierig. Schwierig weil, ich war so aktiv in meinem Land, und hier, ich hab mich gefunden, ohne Arbeit, praktisch ohne/nur Hausfrau [lacht]. So plötzlich Hausfrau [lacht]. Und auch, ja, wie soll ich sagen, auch ein bisschen schlechte Gewissen, so ich bin Ärztin, und ja, ich kann überhaupt nichts machen. Und in dieser zeit hat meine Kollegen so weitergearbeitet und, für mich es hat sehr lang gedauert, diese/so ich bin insgesamt einundhalb Jahre, in Österreich geblieben,ich hab meine zweite Kind in X bekommen, dann es war für mich, fast die Depression [lacht], ja. Und dann, weil ich hab auch von der Studium noch nix angefangen. Und dann mein Mann ist mit dem Turnus, er war mit dem Turnus fertig X, dann sind wir nach meinem Land zurückgeflogen. Und dort ich hab die Möglichkeit gehabt im eine Firma als Arbeitsmedizinerin zu arbeiten. Ja und ja es war eine angenehme Zeit.[lacht] /Wie lang waren Sie dort denn?/ Ein Jahr und halb. Aber dann ich hab diesen Termin für die Nostrifizierung bekommen, und in X sind wir nach Österreich zurückgekommen. Und dann hat es angefangen mit der Nostrifizierung, so Prüfungen, so an der Universität. Und dann hab ich angefangen auch Leute kennenzulernen.  00:52:14-6 

P11: 
Yes, What did you do between 1999 and 2002?
T   			I had babies.
R  			Sorry?
T   			I had my babies.
R   			Oh!! Okay. 
T   			Yes, so I was at home.
R   			So you were a mom?
T    		Yes, and then I was preparing for the exams, reading books.

P11
	The whole package. Yes, and then we had also a clinic, there was a clinic in the hospital, and then we were also doing outreach, we were also going to other clinics that were about like thirty kilometers, fifty kilometers, for we were going to see patients.
R      	And did you have much choice in where you your first job in the Free State, did you want to work there, or where you placed there?
T     	No actually we had a, there were people who were working in that area, then they told us that there was a need in that hospital, so we went and apply and we were internship and then we got the job.
R      		And did you go with your husband?
T      		Yes.

P17
Alors, vous dites que vous voulez faire une spécialisation, savez-vous déjà quelle ?
Ca aussi, c'est un autre problème. Parce que tout que j'ai pensé étant en X, par rapport à ici, c'est pas ce que j'ai trouvé. Donc je suis venue, bloquer dans tout les sens. Mon diplôme d'abord il n'est pas valide ici. Alors pour le valider, ça difficile encore puisque nous sommes en Flandres. Donc pour le valider d'abord il faut apprendre le néerlandais. Il faut faire un examen. Je l'ai valider, mais on a dit qu'il n'est pas équivalent au diplôme d'ici. On m'a même pas dit le nombre d'années que je devais ajouter, on m'a dit juste qu'il faut faire l'examen. Donc il y a cet examen à faire, en néerlandais. Et quand je réussit cet examen, je dois encore faire d'autres années d'étude pour avoir le diplôme de généraliste ici. Donc la spécialisation, je crois que pour cela je dois encore choisir une spécialisation selon les débouchés ici.  Les débouchés de travail, oui oui. Parce que au départ de X, je voulais beaucoup plus faire la santé publique, par rapport à tout cet humanisme dont je vous parle. Et ça aussi c'est vraiment quelque chose qui me tient en cœur. Mais par rapport aux débouchés ici, je dois encore rester un peu pour mes enfants, enfin, mon enfants, les futures que j'aurai. Donc j'ai pensé aussi à faire les médecines du travail. Tout cela parce que j'aimerai bien concilié médecine et contact humaine. 

P17
Et voulez-vous que vos enfants gradient ici ?
Pas complètement. Moi mon objective, c'et quoi.. les enfants vont pas complètement grandir. Peut être si j'ai un métier là-bas, si je gagne un travail là-bas, mon mari déjà il travaille ici. C'est de faire peut être un alternance. Pendant l'année scolaire. Donc un alternance, chacun peut rester un temps avec les enfant ici et pendant les vacances, on rentrent tous. Donc selon son travail ici et selon mon travail là-bas. Si j'ai un diplôme belge, il n'est pas obligé que là-bas je m'engage pour une fonctionne publique. Je peux toujours travailler quelque chose de moi-même. J'ai déjà une association, je veux bien aider la population, donc ça me donne plutôt de.. si je puisse, j'aura mon programme moi-même. Par exemple je fais 6 mois de l'année, je viens travailler ici, en contact avec la population. Et puis 3 mois je de l'année scolaire, je viens en contact de mes enfants. Et puis 3 mois on se retourne. 

______________________________________________________________________

Code: abroad_family constellation {61-0}

P 7: 
Jo'burg it was like all crime, hijacking you know?  Thinking about but I didn't have to because at that time I got married.
R	Okay.
C	And my husband he got a job in South Africa.
R	Yes fantastic and is your husband, where is he from, is he also from…?
C	He is from X.
So your family is here, your immediate family?


P 9: 

Ye, I can imagine. And do you have a family here, besides your husband?
I	Yes, I have my sister, I don't have yet children.  I have my sisters, I have my uncles and we have a Congolese community here.
 

P17: 

Et marier en X, et vivre là ensemble, ça était une option ?
Non non, pas en ce moment. On a pas encore ( ?) de rentrer définitivement, mais on part de temps en temps. Par exemple en ce moment il est là-bas. Pour un mois. Donc de temps en temps on essaie de se préparer pour rentrer définitivement. Donc nous avons commencé à faire des enfants et on va donner le chemin une éducation en bon sens. Et moi aussi entretemps faire une spécialisation. Une bonne formation, et puis, on fait rentrer. 

P17: 

Personnel, je suis fortement liée parce que toute ma famille est là-bas, ma mère, mon père, tout le monde. Je ne suis ici que avec mon mari, et j'ai des oncles à Bruxelles. 

P31: 
And in terms of your future plans, do you see yourself returning?
I	Ja, defiantly I think about it all the time, I think the older I get the more I think its time.
R	And what are your reason?
I	Well I think I just sort of feel you know I've been here all this time and I really would like to spend time with family.  And that's the, for me the biggest one because you know, I'm here I do have friends and I have a life but at the end of the day I would like to grow old, around family, with family around me.
R	Be home.

P38: 

In what ways are you linked to X?

R:	Oh my husband lives in X.  My family, I came here with my son, we travel a lot, we go back to X a lot

P38: 

Is there anything I haven't asked you that you wished I would have asked?

R:	I wrote down some things.  But I think I've talked about the equipment, different work cultures.  Oh one other thing I must say is family support.  Because when you live in a foreign land you don't have your parents to help you with childcare.  I think all that is really important when you come to a foreign land it's…you're on your own…


P45: 

INT: Aaahh your experience of family link, describe your experience of family links.
P: my family, its very difficult you know I am a medical doctor me I can I am working my husband cannot stay here because he cannot find a job especially here in X, he cannot find a job.
INT: mmmhh
P: Yah
INT: Where where is he staying?
P: he is staying in he is moving he is staying in X
INT: Mmmhh the rest of the family, where is the rest of the family?
PF: yah yah, they are there, my son is staying in South Africa
INT: Mmmhh and then the other maybe (murmuring)
P: the husband does his work, my family… parents… mom is in X, my brother and sisters.
INT: how often do you go there maybe for for for events like funerals and weddings?
P: mmm, I didn't go there, I went there once last year to go and visit my parents but in South Africa am going there because my son is there when am off, you know X is far, to go to South Africa you have to take at list 2 weeks you go there you spent time with your son you buy things everything and another day you come here.

______________________________________________________________________

Code: abroad_imp_professional {99-0}~

P 2

Ja, ich war müde, aber das hab ich nicht gespürt, und es war, nicht nur für mich, eine Bereicherung, sondern für meinen Sohn auch. Der war 20 Monate alt als wir [lacht herzlich], dort gegangen sind.  20 Monaten war er und, ein Jahr später sind wir zurück, nach Wien, weil einfach weil, ich hatte die Chirurgie vermisst. Ich wollte mehr operieren und so ein Jahr lang hab ich fast nicht operiert und deswegen wollte ich meinen Vertrag nicht verlängern lassen. 00:12:58-5 


P 2: 
Da war ich unten für zwei, zwei Wochen. Ich konnte nicht länger. Und dazwischen hab ich mich danach niederlassen und so und so. Ich war privat drei Jahre lang, ja ich konnte mich nicht wirklich vertreten lassen und […] 

P 3

I: And you/ do you practice as doctor here? 00:06:53-1 

P2: Here no, I'm not allowed to practice or to work as a doctor because I have to do Nostrifikation, it is a long process you have to put your papers in Gesund, in Health ministry, they accept it yeah, it is real accepted [...?] 00:07:11-9 but I have to do exam for hole medicine subject yeah, in deutsch. And after that they can tell me: no, I have to study more, or no, I can do my, like it is, like, training year or something, its, they call it "Turnus" here, it's almost three years, and it is really very complicated. So i can't do it right now 00:07:47-7 


P 4
P4: Gut, ich bin gerade im Turnus. So ich habe meine Medizinstudium um X angefangen, es war in X, und ja ich habe dort meine Studium abgeschlossen, und ja. Dann habe ich meine Mann kennengelernt, der Österreich ist [lacht], und wir haben dort in Forschungsbereich gearbeitet, und dann so bin ich nach Wien gekommen.Ja, und dann musste ich deutsch lernen weil ich hab überhaupt kein deutsch gesprochen und dann ja und dann die Nostrifizierung und ja. Und dann hab ich langsame zu, in Medizinbereich anfangen zu arbeiten. 00:01:50-6 


P 4: 

Und, gut jetzt so ich vorbereite mich für die Allgemeinmedizinrichtung, so ich weiß es noch nicht wie, wo, ja. Ich werde so mich überall beworben, so hier in Österreich, aber, wir versuchen hier und, ich hab mein Mann gesagt, wenn ich hier keine Stelle bekommen werde, so ich werde versuchen auch im Ausland, ich kann nicht arbeitslos bleiben nach soviele Jahre.[lacht]So viel gekämpft.


P 9: 
To get a position here?
I	To get a post here because I started to work here on the first of October X.  So I stay at home for plus minus three years because I arrived in October X, time to write my HPC exam. X I passed and time to get a job here.  So I started in X, I spent three years.


P11
	The whole package. Yes, and then we had also a clinic, there was a clinic in the hospital, and then we were also doing outreach, we were also going to other clinics that were about like thirty kilometers, fifty kilometers, for we were going to see patients.
R      	And did you have much choice in where you your first job in the Free State, did you want to work there, or where you placed there?
T     	No actually we had a, there were people who were working in that area, then they told us that there was a need in that hospital, so we went and apply and we were internship and then we got the job.
R      		And did you go with your husband?
T      		Yes.

P12

He came January two thousand and four and he passed the May to June exam.
R	Okay well that's quite quick actually.  And in terms of your experience of like the paperwork and the support of the state and things, was it a challenge coming to South Africa or did you find it quite easy?
C	Mmm it was quite challenging because it was not easy for us to get paper especially since we have to, for you to be employed here.  I'm telling you that it shouldn't actually appear but the reality's that you can't get employment if you are not a refugee.  

P15: 

Et quand vous êtes venu…
Quand je suis venu je me suis d'abord occupé de mon traitement et tous. Et quand je sentai que ce serai vraiment difficile pour moi de rentrer chez nous et que j'avais un grand temps à passer ici j'ai dit bon, je vais voir si je peux travailler ici. ..l'époque que je comprenne que c'était pas possible. Avec tous ces problèmes de traiter ses colonnes et.. où on n'accepte pas des médecins avec un diplôme hors Européenne. J'ai compris tout de suite que c'était pas possible.  Alors j'ai essayé de voire les possibilités de reprendre mes études ici. Pour continuer. Parce que j'aime les médecins, je voulais vraiment exercer. Alors je me suis renseigné et j'ai appris qu'il fallait reprendre 4 ans des doctorats. On doit faire un examen où on retenait que 15 personnes pour toute la Belgique. Je me suis dit, oui, je peux passer cet examen mais si on faut reprendre 4 ans, non, j'étais pas d'accord. J'estime que j'ai eu une bonne formation, 4 ans, je trouve quand même que c'est trop. J'ai trouvé que c'était trop, 2 ans, d'accord, mais 4 ans, c'était trop. 
Alors, vous pouvez pas travailler comme une médecine ici..
Non, je peux pas exercer.


P17

Et avez-vous déjà fait le teste de vlir ?
Non, pas encore. Je ne peux pas que pour le mois de septembre. Je n'aurai plus le faire en mars, parce que j'ai rencontré des amis. Elle a déjà fait. Mais bon, mon néerlandais était pas déjà.. Je me suis dit, même si je passe le test écrit, le test oral, je savais rien faire. Donc mieux de me préparer encore. Surtout parce que depuis que j'ai venu, j'ai eu aucun contact, pas de stages, j'ai rien fait aux médecines. J'étais arrivé, j'étais tout de suite enceinte. Donc j'ai pensé, faire la langue, j'ai fait la langue. Et après, quand j'avais accoucher, j'ai encore continué à faire la langue mais ça devenait de plus en plus difficile pour moi. Je me suis dit, alors, je vais plus travailler mon vocabulaire, ça dire parler avec les gens, je veux prendre du temps et revenir à l'école plus tard. Parce que je le fait à l'UCT. Maintenant je fais des petits boulots. Aussi pour gagner ma vie, mais plus pour être en contact avec les gens qui parlent le néerlandais. 


P17: 
Ok. Je pense que nous sommes presque fini, je vais vite regarder mes questions. Est-ce qu'il y a quelque chose que nous n'avons pas déjà discuter, que vous pensez est encore important ?
Moi, si j'ai vite accès à mes études par exemple, j'ai déjà perdu 2 années, donc si j'avais vite accès à les médecines là, vite avoir mon diplôme, je pense que le retour a été encore plus rapide. Je sais pas si vous comprenez.

P31: 

 Well to say I was forced really because the South Africa Health Professional council apparently, well they did, we heard that they were going to change their regulations about foreign doctors, especially from the rest of Africa but essentially foreign doctors being allowed to do post graduate training.  So they were going to be stricter, so you couldn't do it as a, you know cause often when you doing your post graduate specialist training, you employed and you paid a salary.  And you learn on the job, so they were going to stop employing foreign doctors and if you wanted to do your training you'd have to come as a supernumerary position.

P38: 

OK.  And can you tell me the story of your employment after you came to the UK when you decided to stay?

R:	Yes.  When I got to the UK, got the references, are difficult to get in the beginning, even getting jobs is very difficult because in the beginning I had to do care work for, I don't know, for some long time, about four months or something.  I did care work, […].


P38: 

I:	Mm and have you had a clinical registration in the UK?

R:	No that's another issue because I need time to be able to do my PLAB.  When you come in you have to do PLAB if you come from X.  I haven't had time to….yeah….it's the medical schools and some medical curriculum so I haven't had time because it, I would have to get time and money to do that but I think I've given up for that.
