# Supplementary material for: African Female Physicians and Nurses in the Global Care Chain: Qualitative Explorations from Five Destination Countries
Source: PLoS One. 2015 Jun 12;10(6):e0129464. doi: 10.1371/journal.pone.0129464 (PMC4466329; doi:10.1371/journal.pone.0129464)
Supplement: S2 Text — (RTF) [file pone.0129464.s003.rtf]

Original quotes from female nurses - career


P7: Ja ich hatte einige Unsicherheiten einige Freuden aber es verlief gut, ich bin in dem Bereich geblieben, bis zu meiner Schwangerschaft, also 6 und halb Jahre bin ich in dem Bereich geblieben, also eigentlich (?35:09) ohne viele Probleme und so mit ja/

P10

Then I did apply, I sent an application letter with a motivation letter that I came because my husband was transferred here.  

P10: 

I think they just took me in not realising that I am a foreigner.  I think so.  And then I started for a week and then they say but you don't have a permit.  I said I have got and then they said but it is a company's sponsored permit so you have to do your own permit but you have to go back home.  You have to stay at home until everything is sorted.
R	So did you have to do that?
Z	Ja.
R	You had to go back to ?
Z	No, I…
R	Oh you mean...
Z	Ja, ja I was staying at home.  So they gave me a contract, I went to Department, not Department, their Head Office.


P18: 
Puis quand j'ai terminé cette partie là. Entretemps j'avais travaillé, croisé avec mon mari, c'était le mariage. 
On s'est marié et 2 enfants. Il sont tout mariés, j'ai déjà des petits enfants. Mais alors, après le mariage je suis venue ici en Belgique, parce que mon mari était encore étudiant. Je fallait lui rejoindre, ici en Belgique.


P26

After a couple of months I got a post-up in X as a Project Manager setting up the TB post up in X so we moved up there. 

P32: 

And so what happened when you moved to the South Africa in two thousand and five, did your husband stay in X or did you guys come together?
I	He stayed, he stayed. You know, our problem was my husband was doing… his job was paying more than mine.
R	Alright.
I	He was a… I don't know what you call them, he was a bodyguard for the, you know, led by [inaudible] X
R	
P34

Realistically whether I'd have ever done it without my family, I don't know because leaving a daughter on the other end and coming over for a few months would have probably not been so easy but it was awfully tempting. When I mentioned that at home my husband said, "Well if you go why don't we all go?" So it was really a, we had literally sold our house and moved over here within three months of making up our mind.  

P34: 

I kind of decided I wasn't really going to get anywhere clinically and after thirty years of nursing to still be Band 5 and probably with no; I was working part-time so to be honest you're not the next in line for any promotion or any encouragement or anything.  It was kind of a conscious move to move into research, it's quite a new; it isn't new but it's kind of developing as a career for nurses and it's quite nice to get in early so that you can move with it rather than feeling as if you've got this huge big catch up to do. 

______________________________________________________________________

Code: abroad_gender {35-0}

P 5: 
Und deswegen denke ich, auch für die Zukunft in Österreich ist es wichtig, dass man nicht diesen Fehler macht, dass man jetzt sagt, schön und gut, dass wir sagen, wir wollen Matura für die Ausbildung, aber dann mussen wir schauen, was ist dazwischen? Gibt es genug, die an der Basis dann unten die Arbeiten machen? 01:07:21-3 Weil zB eine Krankenschwester hat nie die den Spülraum geputzt, das haben ungelernte Kräfte gemacht, oder eine Krankenschwester hat nie Bettwäsche bestellt für die Station, ja, das hat eine Haushälterin gemacht, die überhaupt keine Ausbildung hatte, die war Haushalts, Haushälterin, die hat sich gekümmert, dass Milch auf der Station ist, dass das Essen kam, dass der Wagen, dass man Essen ausgeteilt hat, und das, und hat die Bestellungen gemacht, was man gebraucht hat für die Station, aber die hat nicht gepflegt! Und die Pflege war dann/ ja man hat die Aufgaben einfach anders verteilt. Und hier ist es so, die Krankenschwester lernt drei Jahre und ist dann zuständig für alles!  01:08:11-6 


P10

Then I did apply, I sent an application letter with a motivation letter that I came because my husband was transferred here.  

P10

So basically two years after arriving it took you to register?
Z	Mmm.  After applying.
R	And the reason you were allowed to register was because your husband was transferred and you were not a refugee or something like that?
Z	Mmm.
R	So what was your status like in terms of paperwork?
Z	I was an accompanying spouse.


P10: 

Okay.  Okay so you don't know yet.  And have you been back to X since you been in South Africa?
Z	Yes we go every year but I have not been like for two years now because I had a baby, so I couldn't travel with the baby.  The baby didn't have a passport yet.  
R	Was your child born here?
Z	Yes.


P23: 
I started off in sort of, it was x, I was, I did my nurse training here in this country at Guy's and I went into Intensive Care and I did paediatric as well as neo-natal ICU so I did those courses and then I went down to Brighton because we relocated down there and I went into the adult coronary care unit at that time and then my husband had an opportunity, he'd already been to South Africa a few years before and had an opportunity to emigrate and so we did.  I went to Johannesburg and we were living in Johannesburg, and worked in the neo-natal unit there.  It was a really, really exciting unit at the time and then I did, following after two years, I did midwifery.  […] we took the decision to come back to the UK and it was whoever was get that job first sort of thing. 

P25: 
 It's sort of, you know, you felt at home than to go back to home to the older restrictions and comparing the nursing care and more specially the way you were treated here in this country as nurses and the way you're treated in South Africa and the money because by this time I had three children who I'd left.  I'd divorced and so I was a single mother really bringing up three children on my own so I also wanted, you know, a bit of money to be able to look after them and then my parents were there to look after my children so I stayed and I'm still here.

P27
P:forget about it… aa that one… so I said okay, I will go to local government,  then I went to the embassy in X, I wanted to find out where exactly where Kalahari was since that was now my choice. So when I got there they were laughing, why do you want to know… a kere that's where I will be working… hee they started laughing… why are you laughing… a no only you are going to the rural area and the tra… problem, transport… there was only a bus once a week.  There will be a problem with transport. And I was leaving my daughter who was doing… to study the… in X, I said let here finish she will come and join me later on. I said since I'm going away I'm going there and the other one… I had a small baby who was about 2 years… now there in the rural areas maybe there are no pre-schools, there are no nursery schools it would be a problem and for my daughter when she comes for holiday for her to go back to school, while she is waiting for that bus which come once a week, she would be like… so I said in that case I will go for ministry of health. 

P29

P: yah, I came here in X03

INT: did you… was there anyone involved in coming to this decision, did you make the decision with your husband or …

P: that time my husband was still alive, we made it together. He came here to do the registration for me.

INT: alright.

P: but unfortunately by the time I came here he was no more.

______________________________________________________________________

Code: abroad_imp_professional {99-0}~

P 5
Und da in X das nicht möglich ist, dass man beide Berufe ausübt, dh man muss sich entscheiden ob man Krankenschwester oder Hebamme ist, und da ich eine Stelle als Krankenschwester bekommen hab, hab ich dann als Krankenschwester gearbeitet. Und war eigentlich nur mit kurzer Unterbrechungen immer in leitender Funktion, entweder Stellvertretung oder Stationsleitung, oder auf einer Intensivstation, in ner Leitungsfunktion. Und jetzt hier nur kurze Zeit ohne Leitung und dann seit jetzt X in ner Leitungsfunktion.

P 5

Und nach zehn Jahren, wenn man ein unbefristete Aufenthaltserlaubnis hat, dann konnte ich einreichen dass ich dann ein deutsche Diplom bekomm. Das ist dann als Krankenschwester, da ist die Anerkennung schriftlich. Weil vorher war nur die mündliche Zusage dass es ident ist und so hab ich als Krankenschwester immer gearbeitet von anfang an, und nach zehn Jahre, als ich zehn Jahre da war hab ich eingereicht, da hab ich die, die unbefristete Aufenthaltserlaubnis gehabt und da ham sie dann, da konnt ich nochmal einreichen und dann haben sie die Urkunde ausgestellt, die schriftliche Bestätigung.  00:33:02-9 

P 5

und wie gesagt ich muss sagen, ich hab immer ein sehr gutes Arbeitsverhältnis gehabt, ich kann das manchmal verstehen wenn andere Schwierigkeiten haben, aber von meiner Person hab ich nie Probleme gehabt.

P 5

I: Was ist für Sie positiv an der Arbeit hier?

P5: Positiv ist das, dass ich obwohl ich Ausländerin bin, auch in eine leitende Position gekommen bin. Durch fleiß und ehrgeiz, dass ich auch was erreicht hab. Und dass ich gerade in diesem Haus, nie das Gefühl hatte ich bin Ausländerin. Weil jetzt ham sehr gemischte Personal, obs Ärzte oder Pflege sind, und da hat man nie diesen Unterschied gespürt. Und auch so von die Wertschätzung der Patienten, ist es ja. 00:43:X-8  00:43:X-8 


P10

From there February, March X I sent a Xx never forgot just thinking that I'll get a job 'just like that' and then I asked my friends who are here already [because I have got another nurse friend over here] she said you apply through the Department of Health, she told me the whole process.  Then I did apply, I sent an application letter with a motivation letter that I came because my husband was transferred here.  That letter went in February X, the same year.  It took a year, I would send emails, ask if they received it, no one acknowledged that they received the application letter, nobody said anything.  You tried to go to their offices they didn't want anyone to come to their offices.  

P10: 
Then I had to take some paper work to Nursing Council again.  This was like starting a process with Nursing Council.  It took about four months because I received that letter from the Department of Health in February.  July then I wrote exams with Nursing Council and then November of X I received the results that I had passed, I had written in July, November I had passed.  February X then I was registered.

P10: 
So basically two years after arriving it took you to register?
Z	Mmm.  After applying.
R	And the reason you were allowed to register was because your husband was transferred and you were not a refugee or something like that?
Z	Mmm.
R	So what was your status like in terms of paperwork?
Z	I was an accompanying spouse.

P10: 
And here you were permanent obviously?
Z	No, I was on an accompanied spouse permit.  So now I had to start looking for a job, I thought it was easy after I had my registration.  I started hopping around every government hospital looking for a job and then I couldn't get anything.

P10: 
What difference does it make?  I mean from your experience, if you've got all the right papers and things, what extra responsibility would they have?
Z	I think the paper work because there is also paper work after they employ you.  Like here, I was employed in October and I started work in April, just this month.

P10


Wow!  Just to get the paper work!
Z	Just to get the paper work through because the department of Health, after writing exams, you tell the Department of Health, the Department of Health gives you a letter that now you can go for a job.  So you move around with that letter and then they will give you a contract.

P10
So obviously the facilities don't want to take on that burden?
Z	Because when I was employed here what happened is.  I think they just took me in not realising. 
R	Ja.
Z	I think they just took me in not realising that I am a foreigner.  I think so.  And then I started for a week and then they say but you don't have a permit.  I said I have got and then they said but it is a company's sponsored permit so you have to do your own permit but you have to go back home.  You have to stay at home until everything is sorted.


P18: 

Alors, vous êtes venue en France. Vous avez fait les études de 6 mois là-bas ?
Oui. J'ai fait les études de 6 mois en santé publique. Et puis alors quand j'ai terminé, j'ai travaillé aussi au même temps. J'étais engagé comme aide soignante dans un maison de repos. Et entretemps, j'ai entrepris aussi d'autres études en peu spéciales. C'est quelque chose qui s'occupe, qui soigne à base des plantes.


P18: 
Donc votre diplôme est pas reconnu ici ?
Oui, c'est ça le problème. J'ai essayé de chercher des informations, faire l'équivalence mais le réponse était toujours il fallait refaire la dernière année, ou .. Faire encore des stages.. Tout ce qu'on a fait là, il faut refaire. J'ai dit : c'est pas possible. Et c'est comme ça que j'ai accepté le travail comme aide soignante. 
Mais c'est pas facile e, dure, dans des maisons de repos. Et c'était difficile d'avoir un séjour comme ça, et j'étais obligé de reprendre mes études, mais cette fois si, c'est plus en domaine médicale. […]. Et quand mes papiers était bien en ordre, j'ai commencé à chercher du travail alors. J'ai trouvé d'abord dans une maison de repos, mais comme je ne l'aime pas vraiment. J'ai fait un effort à faire des intérims, et maintenant je travaille dans l'hôpital de revalidation.
J'ai aussi une association. J'aime bien. Je fais l'accueil des migrants. C'est là où je parle. Parce que la théologie, c'est pas seulement parler du dieu, c'est l'amour, les prochaines. Mon association c'est fraternel et aide développement. Donc on accueillit les migrants.

P23

I started off in sort of, it was X, I was, I did my nurse training here in this country at Guy's and I went into Intensive Care and I did paediatric as well as neo-natal ICU so I did those courses and then I went down to X because we relocated down there and I went into the adult coronary care unit at that time and then my husband had an opportunity, he'd already been to South Africa a few years before and had an opportunity to emigrate and so we did.

P24

 I'm the Ward Manager now and this morning for instance, I went into the handover room and there wasn't a chair but there's two first year students sitting there like this, you know, and I just want to shake them and say, "You need to wake up."  Anyway so I had to go out and get myself a chair which would never happen in South Africa, it's just a question of respect you know.  As a first year student I didn't even sit with the nurses, I'm not saying we should go down that route, I mean that's not what I'm saying, it's just I think in South Africa there's much more respect for the profession which I don't find in the UK.  And that would be one of the reasons why we'd go back.  No-one in South Africa will call you in your first name, you know, a patient.


P25: 

And you told me a lot about your different employments coming to the UK but I was interested to hear about the teaching.  Can you tell me a bit about how you went from nursing to teaching?

R:	Teaching yeah.  No, no but this was now in this country.  I was working in…I've always been, even in South Africa, I've always been interested to teach people like, you know, when there's a new person coming, you know, I was always the chosen one to make sure that they know the ropes and so I was; I had the teaching role not like a teacher but I was teaching in the clinical setting.  But when I came over here I think my, you know, my manager's here, they were not called matrons, they were like nursing officers or something.  They recognised that I was a good teacher in the clinical setting so then I was seconded to go and study, you know, nurse education in the University of X to be a teacher of nurses.  So when I say I was a teacher I was still teaching nurses.  So I ended up teaching nurses in X.

P25: 
When we first came here the….we, it was actually difficult here….as I've said by the time I left South Africa I was a senior nurse so you come to a new country whereby, first of all, people really treated you as an underdog really.  I mean I don't like to say but it was the affect that because of your colour; because you came from Africa the third world so they thought that South Africa, we were like the third world.  Somebody would ask me, "Oh where did you buy your clothes, did you buy them at the airport?  Do you wear clothes in South Africa; I thought you wear, you know, those kind of things."  Some nurse would ask me, "Oh," you know I did temperatures, "Oh do you know how to check the temperatures?"  "Yes," "Oh where did you learn how to check temperatures?"  And I was already a trained nurse in here when I came to this country and, "Oh so you know how to check temperatures in South Africa?  The other one they were like the incubator oh "Have you ever touched this incubator" or you know, "Have you seen it before?"  You know those kind of things so god, you know when

P25: 

No in fact even a vaginal, an urethra they've never done that.  And then in South Africa it's done by a second year student nurse and you….they can't put a nasogastric tube and it's a trained nurse.  In South Africa your first year or you know, you can put a nasogastric tube; you know those kind of things.  So we've came here with all that knowledge, charging you know that when we come here, I'm doing this, I was showing them that I can do this and then when you come here, no that's it, you cannot even give paracetamol.  You are, they are even questioning that you can do a, b and c.  So that really did like you know, we, it breaks like in the speed then just halted us and there's, 'Oh my god, this is a different country.'  Then in the end you decided that you pretend that sometimes you don't know how to do things simply because you are either lazy to do it or you say, "OK you do something now," I'll say, "I don't know how to do it."  You know that kind of thing and then you watch them doing it the wrong way and then that you either kept quiet or you say, "Oh by the way I can do it."


P26

When we came over I was waiting for my registration to come through so it was a case of working as an HCA, working through X away from my husband and I didn't cope with that either so I came back and that was when I started working as an HCA at the local hospice through an agency. 

P27

INT: so he was involved in the decision?

P:he was involved in the decision, so that's how I came in X; I came here to register as a nurse. It took me 2 years because it's not all that easy. They really have to prove that you are a nurse where you come from. So it took 2 years, after registering that's when… on the day… I used to come, to check how far… to come to check how far. The day when the registration was complete I was here, I was already here, they were just laughing, they said how did you know by hour later… we haven't posted it, how come you have come, no you know that I have been checking on you. You know today everything is okay just bring that much, this amount to register than you can go and apply. You to the ministry of health, you can go to the local government. So I applied both and 3 months time I receive both jobs, now it was to choose which one now ministry of health or local government.

P28

INT: ok. So now, living in Botswana itself, can you tell me the story of your employment after coming to Botswana? Where have you worked, in what area.

P: when I came, first I was a full-time house wife before I got the employment. I stayed home. Because they didn't come with my papers from the nursing council… so at the end of the first contract I registered…

INT: so where did you work before you came?

P: I worked… I worked here. I started in November 'x
INT: oh so you worked for local government as well.

P: I started in Kgalagadi I think it was less than 2 years. Then I moved to Kalahari North

INT: was your husband working there too?

P: No, he was in X.

P30
Eventually we coped.  We had difficulties, you know, and those from being, coming from another country and you know as nurses you can't practice if you don't have your practicing license…

P30: 
Tell me.
I	… ja, they need documentation, it's another process altogether because they need proof that you are registered back home, they need a letter from the university that you apply and it's not there and then it's a process [inaudible]  Ah, it's really another process.
R	So how long did it take you to register with the nursing council?
I	Actually in the first year we didn't manage, we only managed in the second year because you first have to go through the Department of Health then, ja, the Department of Health gives you a letter which should be an attachment to the required documents to submit at the SANC, the South African Nursing Council, that's when they issue.  So we are only issued in the second year.

P33: 

So when we travelled to South Africa the only challenge we had was our registration with the South African Nursing Council.
R	Okay.
I	It took us almost two years to be registered.

P33: 

And because we didn't start that process in x, we started it from here but still I didn't expect that while we are within South Africa we should have that long, long…
R	Wait.
I	… waiting because everything was readily available and so there was no justification for that long process.  And so we…

P33

And how did they register you, did they look at your qualifications to give you an equivalent in South Africa or…?
I	Yes, they take our certificates, they go to SAQA and they compare the qualification and then they put us on the right grade with South African standards.  That process went on well but now to get feedback from the nursing council it's hell, it was not easy until we had to travel to and from, to and from and it was costly and also like mentally we were disturbed because we could not do our clinical practice because we needed that registration process first.

P34: 

And tell me about your travel before then and when had you been working here before.

R:	So in the late eighties I'd come over here to travel.  I'd run out of money and got a job as a carer in a home for the elderly.  It was alright, again not really my kind of job.  I got on well with some of the residents.  I do like to do jobs where you get a little bit of something back and some of them you didn't at all because they were quite brain degenerated really, not quite with us and that's a really difficult environment to work in. 

P34

I kind of decided I wasn't really going to get anywhere clinically and after thirty years of nursing to still be Band 5 and probably with no; I was working part-time so to be honest you're not the next in line for any promotion or any encouragement or anything.  It was kind of a conscious move to move into research, it's quite a new; it isn't new but it's kind of developing as a career for nurses and it's quite nice to get in early so that you can move with it rather than feeling as if you've got this huge big catch up to do. 


P36: 

I went into a private hospital to work as a, just in a general ward, high dependency ward, general ward.  Just putting up a drip, I couldn't, I didn't have the confidence to do that, and making decisions like, you know, they expect you to do it because you're a Sister, you've got epaulettes.  But you can't, you're like here were you've not really had, I mean back when I was still working in the hospital in x you didn't just make a decision, you know easily, that you would have made back in X, so I felt I couldn't put myself through that, through that or in having to work again and work my way up again.  

P37: 

Sure, sure.  And you said that you thought you would have emigrated anyway? 

 R:	Yeah, yeah we would have, yeah.

I:	And it's, when did you come to that decision do you think?

R:	Well I think it was after the two years that we were here and we were offered to extend our, or he was offered to extend his contract.  We actually weighed up the standard of living here and the standard of living there and based on the crime rate we decided actually, you know what, it's better to just stay here.

P37: 

Yeah.  Can you tell me the story of your employment after coming to the UK?

R:	Yes.  I actually, we lived, we live in X and my son went to the X school and X school's a boarding school and though my first few months, the plan was to settle in the family and the school approached me and said that there was a nursing staff, a nursing member that had gone off with breast cancer and would I do a six month locum for them at the school, at the school.  So I went and worked up at the school.


P37: 

And after working in a school?

R:	I then went to work at X hospital.  It was just the other side of the hill so I used to drive past it regularly because it was set well back and very nice grounds.  I used to think it was a psychiatric hospital because that's what South Africa's like and decided to just pop in there one day and see if they had anything and they, well I started working for them then and it was in heart screening because I'd done occupational health and all the screens for them and just progressed from there basically.

I:	Yeah.  And what other types of problems have you had?

R:	Well I worked at X for five years.  I set up their x department and I think because I was into setting up clinics in South Africa, they then built on a wing and commissioned me to the wing to help them with the set up and layout of that wing and then the X Group [private] approached me and asked if I'd go and set up some nursing clinics for them.

P37: 

No I don't know.  Do you see yourself; you've already touched on this a little bit, do you see yourself returning to x?

R:	We have thought about, my husband would love to go but; he'd go tomorrow if I said yes and that's me saying no.  Opportunity for myself permanently, first of all being white, secondly over fifty years old, I don't think there's any opportunity at all, and I was very fortunate to be able to bring my kids up and work very part-time.  I never ever thought of a career, got here and my career took off.  I would never have had that opportunity in x to be honest.

P39: 

Two – working in the ward with somebody who'll tell you that, "Oh you are a registered nurse?"  No but I'd been a carer in this ward for more than ten years so don't come with an attitude of thinking you're going to run this ward.  I'm in charge here, that's the most fascinating thing I ever experienced.  Then where there's an exchange of roles that because I'm a nurse and I qualified in South Africa, together with the fact that I'm black, I cannot work as a registered nurse with the healthcare assistant who's been there for ten years.  I have to do her job then she will do mine.  Hang on who will account here?  It was one of the toughest things but I had to use a positive approach although it was tough because the time I came there were no human rights, there was no act to back my abilities and my understanding of what's acceptable and not except that I would rely on, 'Hey you know there's what is called, the nurse, the governing body for the nurses, if I don't do this, I'll be in trouble'.  Then she'll say, "Even the ones I worked with before, they were trained so don't come with an attitude."  Ah that was the hardest thing in this country so. 

P39: 
 Then when you worked with them then the conflicts and the targets, then you just have to understand that yes you are now in another planet.  

P46
So do you feel like there's more independence here or in x?

R:	I think x health care services are well organised and as a professional; because as a nurse you have worked hard, we have undergone intensive training so we have acquired a lot of knowledge so you are able to practice what we have studied.  Unlike in UK you can be here as long as you are not a manager, you are always dependent, you are being told, we are like a remote control, we have got no input in the policies, you have got nothing.  In x the policies, they depend on nurses who are able to change policies, to formulate policies, to evaluate policies – something we don't do in England - all the time I've been told.

P46: 
 In UK if; that what disappointed me, if you are a foreigner, you're a foreigner, it's difficult for you to move even if you know, like here I was a; when I came here I was much experienced but if you're a foreigner there isn't much for you and they don't tell you what is expected of you.  

P46: 

But always here even a minor thing you…they will write an incident so it makes you not to be free and it makes you, what is it, at times because there are many X, there are many things that you don't do in UK even if you know it but from home, like small, small things.  Like taking off bloods, you are not supposed to do that until you are trained, all those minor, minor procedures which were being done by the students in my country who wouldn't do it here.  So it was a bit depressing and a bit; and you do all those data things like, oh not data, I won't say those minor activities which were done by the junior members at home.  So there was nothing that is motivating you as a nurse.  But now what I've found here in UK, all they are concerned about is money, we work for money. 

P46: 
And what influenced your decision to move?

R:	For me it was; I thought that the payment is much more than in x but when I came here it was not, it's almost the same.  For you to earn more in UK you have to work hard, long hours, you have to maybe have one, what is it, one day off or you don't; so you have to work hard.  So that is the point that when I thought that the payment was better and also I came because I wanted to study.  That is it, that's what made me to come to this country.

P46: 

Is this racism at the workplace or just?

R:	At workplace, everywhere there is racism here.  Even the patients themselves they're racists.  Even doctors themselves, they are racist.  So you have to prove yourself  X professional, we have to prove.  Like taking the orders, the doctor will go to a white nurse or your white healthcare assistants to give orders instead of coming to you because you are black.  So racism is too much.
