# Supplementary material for: African Female Physicians and Nurses in the Global Care Chain: Qualitative Explorations from Five Destination Countries
Source: PLoS One. 2015 Jun 12;10(6):e0129464. doi: 10.1371/journal.pone.0129464 (PMC4466329; doi:10.1371/journal.pone.0129464)
Supplement: S3 Text — (RTF) [file pone.0129464.s004.rtf]

Original quotes from interviews with female physicians - Difficulties

P 4

P4: Schwierig. Schwierig. So, ich könnte keine deutsch sprechen, und so, und dann so ich hab im 2000 hab ich meine Tochter bekommen, und ja es war für mir schwierig. Schwierig weil, ich war so aktiv in meinem Land, und hier, ich hab mich gefunden, ohne Arbeit, praktisch ohne/nur Hausfrau [lacht]. So plötzlich Hausfrau [lacht]. Und auch, ja, wie soll ich sagen, auch ein bisschen schlechte Gewissen, so ich bin Ärztin, und ja, ich kann überhaupt nichts machen. Und in dieser zeit hat meine Kollegen so weitergearbeitet und, für mich es hat sehr lang gedauert, diese/so ich bin insgesamt einundhalb Jahre, in Österreich geblieben,ich hab meine zweite Kind 2001 bekommen, dann es war für mich, fast die Depression [lacht], ja. Und dann, weil ich hab auch von der Studium noch nix angefangen. Und dann mein Mann ist mit dem Turnus, er war mit dem Turnus fertig 2001, dann sind wir nach meinem Land zurückgeflogen. Und dort ich hab die Möglichkeit gehabt im eine Firma als Arbeitsmedizinerin zu arbeiten. Ja und ja es war eine angenehme Zeit.[lacht] /Wie lang waren Sie dort denn?/ Ein Jahr und halb. Aber dann ich hab diesen Termin für die Nostrifizierung bekommen, und 2003 sind wir nach Österreich zurückgekommen. Und dann hat es angefangen mit der Nostrifizierung, so Prüfungen, so an der Universität. Und dann hab ich angefangen auch Leute kennenzulernen.  00:52:14-6 


P 4: 

P4: Ich weiß noch nicht. Nein, ich hab mich beworben,[…]  gearbeitet, so ich hab dort mich beworben, habe, weil ich Kinder habe und, so ich habe nie eine Stelle dort bekommen. Und dann ich war in […] Spital, ich hab mich dort beworben, weil dort gibts eine […], ich war dort sieben Monate, habe, trotzdem sie haben mich auch nicht genommen. Weil keine Stelle war und gut. 

P 7: 

And at that time I was having so many choices, I could write an exam for France to go and further my study, or my brother was already this side.  So I was like either go to South Africa where I can work as a doctor, than go to Europe where you have to do other things there you know.
R	To study more.

P 7

C	So what did you do in Swaziland?
R	Yeah I did work in Swaziland for four years, I got a contract because here you're supposed to do an exam and that time they were not doing an exam.  So in Swaziland it was just a matter of being patient to submit your paper and work and wait.  So I was trying to do some local stuff and that you know.

P 7

R	Yeah okay and any other reasons why you chose to leave, was it a difficult decision to make?
[…] R	Yeah, yeah so did you learn English on the job?
C	Yes it wasn't very easy. 	
R	I imagine it's difficult. 
C	It was very difficult. 

P 7: 

Yeah you don't know.
C	Because nobody told me you know it's quite different because here in South Africa there are also other aspects that we have here but you know it's not that easy also. So in terms of x and them, in terms of you know in crime things you know when you hear what is happening to other people, so you wonder these things we didn't experience back home you know.

P 9: 

Okay, can you tell me more about your experience when you came to South Africa?
I	When I came to South Africa the first challenge was the language. I had to practice my English. The one I have learned when I was in high school and after that, I was supposed to write the exam, the HPC exam. And it took me one year to pass my exam.
R	Is that mostly because of the language? 
I	Ja, when you study those things, it not so difficult, it was the language now.

P 9: 
And what was that experience like going to home affairs?
I	It was bad. It's the worst experience I have.
R	Can you tell me more about it? What happens?
I	You know you arrive here as a medical doctor. Of course seeking future but you are somebody qualified. No, we spent the, we spent the night outside. I don't know if you have ever been to Pretoria home affairs? You see what happen there. You wait outside, maybe for two nights, two nights. And then, if you are fortunate then they call you. For me it wasn't so bad cause I was a young lady. They start by a lady and then when we enter there. They ask us about, we have a short interview and they give us the [inaudible] sticker. Which was renewal after two or three month.  

P 9

Did you have, did you go with your husband or he was already working, so what?
I	He was not working, he was also working for him. You know it took also time for him.  To start working. […] So we were going to the home affairs fortunately, by the grace of God we had the paper within two years and we came back to the foreign work force telling them, look now we having our paper can't you please give us a job?  And then they give us the job but it took time.

P 9
No, they say, they have to do all this stuff. By the home affairs they can't. We explain to them many times that we if look, you can't renew us, the permit's, three months before because the GCC it takes time. If you can't actually renew us three month earlier so we won't run short of contract or salary.

P 9

Ja, in the whole district?
I	In the whole district. All of us, starting by […] after doctor, after Doctor […] all of us, we are foreigners but it seems like also, upstairs people are not happy. Because there is a big shortage, how many times there are advertised for the post? People are coming but they are foreigners and they can't employ them.

P11

No I was staying in Johannesburg then after writing the exams [in x ], then waiting for the whole process because the qualification needed to be verified in Philadelphia. After waiting for the whole process which took quite long, so I started working in x then I started in the Free State.

P11

Do you find it kind of smooth sailing or was it challenging to get your papers in order and all that kind of stuff?
T       	To go for papers, you go there is a system that is there, you need to go to the home affairs, then they give you a paper for two years, you renew it, then after five years you apply for a permanent residence. So we went through the whole process right now of having my permanent residence.
R     	And which passport do you hold, do you have a South African passport or a...
T       	No I am not yet having a South African passport because you need to have a citizenship.

P17

Alors, vous dites que vous voulez faire une spécialisation, savez-vous déjà quelle ?
Ca aussi, c'est un autre problème. Parce que tout que j'ai pensé étant en X, par rapport à ici, c'est pas ce que j'ai trouvé. Donc je suis venue, bloquer dans tout les sens. Mon diplôme d'abord il n'est pas valide ici. Alors pour le valider, ça difficile encore puisque nous sommes en Flandres. Donc pour le valider d'abord il faut apprendre le néerlandais. Il faut faire un examen. Je l'ai valider, mais on a dit qu'il n'est pas équivalent au diplôme d'ici. On m'a même pas dit le nombre d'années que je devais ajouter, on m'a dit juste qu'il faut faire l'examen. Donc il y a cet examen à faire, en néerlandais. Et quand je réussit cet examen, je dois encore faire d'autres années d'étude pour avoir le diplôme de généraliste ici. Donc la spécialisation, je crois que pour cela je dois encore choisir une spécialisation selon les débouchés ici.  Les débouchés de travail, oui oui. Parce que au départ de X, je voulais beaucoup plus faire la santé publique, par rapport à tout cet humanisme dont je vous parle. Et ça aussi c'est vraiment quelque chose qui me tient en cœur. Mais par rapport aux débouchés ici, je dois encore rester un peu pour mes enfants, enfin, mon enfants, les futures que j'aurai. Donc j'ai pensé aussi à faire les médecines du travail. Tout cela parce que j'aimerai bien concilié médecine et contact humaine. 

P17

Et avez-vous déjà fait le teste de vlir ?
Non, pas encore. Je ne peux pas que pour le mois de septembre. Je n'aurai plus le faire en mars, parce que j'ai rencontré des amis. Elle a déjà fait. Mais bon, mon néerlandais était pas déjà.. Je me suis dit, même si je passe le test écrit, le test oral, je savais rien faire. Donc mieux de me préparer encore. Surtout parce que depuis que j'ai venu, j'ai eu aucun contact, pas de stages, j'ai rien fait aux médecines. J'étais arrivé, j'étais tout de suite enceinte. Donc j'ai pensé, faire la langue, j'ai fait la langue. Et après, quand j'avais accoucher, j'ai encore continué à faire la langue mais ça devenait de plus en plus difficile pour moi. Je me suis dit, alors, je vais plus travailler mon vocabulaire, ça dire parler avec les gens, je veux prendre du temps et revenir à l'école plus tard. Parce que je le fait à l'UCT. Maintenant je fais des petits boulots. Aussi pour gagner ma vie, mais plus pour être en contact avec les gens qui parlent le néerlandais. Pour mieux m'exercer. Parce que je suis ici, je ne suis pas assez intégré pour avoir des amis qui parlent le néerlandais, non. Je n'en ai pas. J'ai que des amis qui parlent le français.


P31

Its was around ninety six and that's when that discussion started and so, so I was worried that you know, I'm sort of trying so hard to get into pediatrics programs and getting all these letters saying, sorry we giving first opportunities to South African's and I understood that completely but you know.  Pediatrics was highly competitive and so I decided to try public health, you know, still another specialty that I was interested.

P31
And be at home and feel, I mean it hasn't been easy in South Africa, you know all these years leaving in this country they still a sense of you're an outsider and not so much in the social circles I move in but generally the environment in South Africa, hasn't been that friendly.  And I feel like I've had enough.

P31

And I'm not suggesting that people should embrace me and be wonderful and everything and it not even just about me, its generally the social environment in South Africa.  The, you know, the sense that you know you can't do anything unless you have a green ID book which you can't get until you've lived here at least five years and have long list of motivations supporting you and even then that law only came about a few years ago.  

P38: 
Is there anything I haven't asked you that you wished I would have asked?

R:	I wrote down some things. But I think I've talked about the equipment, different work cultures.  Oh one other thing I must say is family support. Because when you live in a foreign land you don't have your parents to help you with childcare. I think all that is really important when you come to a foreign land it's…you're on your own…


P45
INT: Mmmhh, and then anything that you found difficult that's where what you wanted to talk about there…
P: yah yah. That one is the the patient aahh you know I was thinking you know I was thinking maybe its just the same. The patients everywhere they are the same, but here in maun the patient are not the same, the patient they can insult you, they can even beat you, they can even do funny thing on you and it was very difficult for me everyday I just then went..

______________________________________________________________________


And the oral exam.
I	Yes, it was the language; it was like I came in 2006, I passed my exam in 2007 then that time my husband was already working here. And I was now looking for a job.  Of course we tried at X but they were having like a memo saying they don't want foreigners, they can't work in X.
R	Why was that?  What was the reason, I don't remember?
I	They say foreigners they don't have, because Gauteng, Cape Town and Durban, foreigners, we have to go to the rural areas.

P 9
Yes, I am very grateful to Doctor X and X they were fighting, fighting with the Foreign Health Department in Pretoria.  They were busy calling, motivating, that why it took two years.  That time I remember it was difficult, now I don't know things how it happen but that time I remember it was very difficult, the say it was impossible.  I remember one day, one lady from the foreign work force called me she say “Why you have to wait here? Do you have to work here in Gauteng?  Because we suggested to have second choice.”  I say “I don't have any second choice, I want to work in Johannesburg because my husband is working here” […] 

P 9: 

Last year, I applied for a post in family medicine. They didn't take me, I don't know why. Let me tell you, they say when I ask, why you didn't tell me, take me, they say first of all because I am a foreigner.  And I don't know if you know, in that program, in family medicine there is a lot of foreigners, so I have been told that, I heard like that, they wanted also South Africans to come in. 

P 9: 
They call her for interview, she went, she passed but she didn't, she never came. She say “I am not ready” So they, I apply for family medicine they didn't take me. Okay, it's fine, maybe I am going to apply again next year. I have to study because we don't qualify for Gynae, Surgery….

R	Why not?
I	Because we are foreigners.  We are, we are, let's say because we are refugee.  Before to apply for other departments you have to have at least the permanent residents which we don't have.

P 9: 
Because you know, I don't know who is that doctor, South Africa doctor working in the hospital, we leave the hospital and come to primary health care. I don't know. I was, even our interns, if you ask them after there, there postage in the location. When you ask them, do you want to come back to the primary health care? They say no because there is a lot of work. Contrary what people think in the hospital they work more that in the clinic. It's not true, in the clinic, in the primary health work, where I've, we work more than those who are in the hospital because in the hospital, when you do your round, one round in the morning, if you don't have clinic, you don't have surgery, you can leave. It's not officially but it's what happening. You know but here in the clinic, you can't leave because there is no one around, you have to push the queue, push the queue, push the queue.  So South African you don't like that, it's a lot of work.  So foreigners we take that, we are filling because we need the job.

P15: 
Et quand vous êtes venu…
Quand je suis venu je me suis d'abord occupé de mon traitement et tous. Et quand je sentai que ce serai vraiment difficile pour moi de rentrer chez nous et que j'avais un grand temps à passer ici j'ai dit bon, je vais voire si je peux travailler ici. ..l'époque que je comprenne que c'était pas possible. Avec tous ces problèmes de traiter ses colonnes et.. où on n'accepte pas des médecins avec un diplôme hors Européenne. J'ai compris tout de suite que c'était pas possible.  Alors j'ai essayé de voire les possibilités de reprendre mes études ici. Pour continuer. Parce que j'aime les médecins, je voulais vraiment exercer. Alors je me suis renseigné et j'ai appris qu'il fallait reprendre 4 ans des doctorats. On doit faire un examen où on retenait que 15 personnes pour toute la Belgique. Je me suis dit, oui, je peux passer cet examen mais si on faut reprendre 4 ans, non, j'étais pas d'accord. J'estime que j'ai eu une bonne formation, 4 ans, je trouve quand même que c'est trop. J'ai trouvé que c'était trop, 2 ans, d'accord, mais 4 ans, c'était trop. 
Alors, vous pouvez pas travailler comme une médecine ici..
Non, je peux pas exercer.

P31

Okay and can I ask about that process, a little bit more.  So obviously ninety four things were changing quite radically and so, so how easy was it for you to come here?
I	Surprisingly well, I didn't realize then until, later on with new, as developments happened.  It was actually easier to come to South Africa soon after ninety four than it is now.  As a doctor in Africa.  Because actually at the time, you know it was very simple, there was an exam, South African Medical Council offered an exam, you wrote, if you passed the exam then you were eligible to register and you could apply for a job and assuming you were given a job offer, home affairs will then you know, give you a work permit.

P31: 

 Well to say I was forced really because the South Africa Health Professional council apparently, well they did, we heard that they were going to change their regulations about foreign doctors, especially from the rest of Africa but essentially foreign doctors being allowed to do post graduate training.  So they were going to be stricter, so you couldn't do it as a, you know cause often when you doing your post graduate specialist training, you employed and you paid a salary.  And you learn on the job, so they were going to stop employing foreign doctors and if you wanted to do your training you'd have to come as a supernumerary position.

P31

Just perceptions or receptions?
I	Well sometime reception is poor, sometimes people just have certain perceptions about Africans from the rest of Africa. There's, there's no sense of, one doesn't all, I supposed I, you know people who gone to, it's probably not unique to South Africa, I'm sure it's the same everywhere. You know the sense of other, there's a sense of you're another person.  I know when I moved to South Africa people actually thought that, oh god there's some much racism at least now the black people are in the majority and it will be fine but there's no sense of, your still, okay you're black but your African and so.
P31
The university is open and welcoming, I mean that to me has been the reason I've stayed, you know.  But from a social sort of environment point of view I think there are issues about, I don't know about how other Africans, other Africans from the rest of Africa experience it but I know my experience resonates with many of my fellow X.  It's an ongoing struggle and one just, you sort of get tired of it and think it's time to go back home.
R	Sure.
I	And, and so I feel like you know if I'm going to grow old I'd rather do it in my own place.

P38

And another challenge I found when I came here; in X I went through very easily to the top but then when I got here it's really difficult and I think it's because of the culture or my accent or I mean, there's some things you just feel you're not going through, sailing through easily, it's really hard if you're coming from somewhere else into this system, the UK system, it's really hard and one other thing I find – I mean I think it's all the culture and when you haven't grown up here, so when people talk about something which is general it's not general for you at all so at times it's really frustrating  and difficult, yeah.  

P38: 

Had you been, any colleagues or friends gone down that route before?

R:	Yeah I think when I was doing my Masters somebody from Africa, I think she was from Ivory Coast; she'd given me an agency, an agency that offers health care work.  Yes she was from Africa.  One other expense was this visa renewal and things when you're here, it's really difficult and expensive and the fees just keep on going up, it's just too much.  Yeah so some of those issues you have, you can't talk about them in the team, nobody, some people wouldn't know what you're going through as a foreign person working in this country because at times I hear my colleagues say, "Why do these foreign doctors come?  Well I don't know they  just do any work and do healthcare work."  I tend to fee, it's really terrible, but I know what it is but some people here just don't.  It's just, and there's somebody in particular who just keeps on making all those comments about foreign workers who  come and just do….which I find very unfair and at times very personal as well.

P45: 

I was thinking maybe I explained to the patient she will un she will understand me, the following day she went to she went to tell the matron or the the the owner of the the ward, the department I chase her away I beat her I did funny thing with her. I was upset, I say hhh how can I beat the patient she was very sick. I could even touch her how can I beat her aaoo. And then I feel like why she behave like this when I was trying to help her she needed O², she needed oxygen in the office where there were no oxygen. How im a going ot get that oxy oxygen to her, it wasn't there but where I feel like I told her away. There are many examples many, there are a lot I could not tell could not tell them here, they are a lot.

P45
INT: like maybe I don't maybe the working conditions aah
P: the thing is just if they can teach the patients to respect us doctors, doctors and nurses. We know we are foreigners, but we are giving good service here if they could just respect us I will be happy to work here in Botswana, even to stay here forever. But while the people the patients are not respecting you they can even insult you they can do funny thing on you, you know, there are other times when you go home you couldn't even sleep you were just thinking why that patient behave like this why.

P45: 
can you suggest …
P: the thing I will just suggest if they can consider us foreign doctors or foreign nurses especially here in X. When you are going to immigration you see the way you will not like, you will not like to stay here

______________________________________________________________________

Code: abroad_racism {16-0}

P 7: 
 We were like three or four people and they just search him and they got money from him, take his luggage and they went home and they like lock us inside.  So we had to scream and call the police and the police were like just the street behind but it took them I think forty five minutes to come there, so we like [maybe because we are foreigners you know] we just feel a bit neglected at some point.  


P 9: 
Because you know, I don't know who is that doctor, South Africa doctor working in the hospital, we leave the hospital and come to primary health care.  I don't know.  I was, even our interns, if you ask them after there, there postage in the location.  When you ask them, do you want to come back to the primary health care?  They say no because there is a lot of work.  Contrary what people think in the hospital they work more that in the clinic.  It's not true, in the clinic, in the primary health work, where I've, we work more than those who are in the hospital because in the hospital, when you do your round, one round in the morning, if you don't have clinic, you don't have surgery, you can leave.  It's not officially but it's what happening.  You know but here in the clinic, you can't leave because there is no one around, you have to push the queue, push the queue, push the queue.  So South African you don't like that, it's a lot of work.  So foreigners we take that, we are filling because we need the job.

P11: 

just in your life in general?
T      	I didn't experience it as, personally, but I remember there were, there were that time of xenophobia in the hospital, we were asked to stay back, to stay home and not to be exposed.
R     	Ja I mean that was 2008, that was quite a challenge, ja, I was just wondering if that came up.

P31: 

Just perceptions or receptions?
I	Well sometime reception is poor, sometimes people just have certain perceptions about Africans from the rest of Africa.  There's, there's no sense of, one doesn't all, I supposed I, you know people who gone to, it's probably not unique to South Africa, I'm sure it's the same everywhere.  You know the sense of other, there's a sense of you're another person.  I know when I moved to South Africa people actually thought that, oh god there's some much racism at least now the black people are in the majority and it will be fine but there's no sense of, your still, okay you're black but your African and so….  From the rest of Africa.

______________________________________________________________________
