# Supplementary material for: African Female Physicians and Nurses in the Global Care Chain: Qualitative Explorations from Five Destination Countries
Source: PLoS One. 2015 Jun 12;10(6):e0129464. doi: 10.1371/journal.pone.0129464 (PMC4466329; doi:10.1371/journal.pone.0129464)
Supplement: S4 Text — (RTF) [file pone.0129464.s005.rtf]

Original quotes from female nurses - difficulties


P 5: 

P5: Wenn ich zu sagen hätte, ja. Hab ich leider nicht (lacht), aber. Ich denke einfach, die Regierung weiß gar nicht, wieviel Potential da ist, wenn man jemand aus nem fremden Land aufnimmt, die schon in seinem Land gelernt hat, weil da war es schon so, dass ich mich ein bissl diskriminiert gefühlt habe, nachdem ich 17 Jahren in x gearbeitet hab und wenn man die Zeitspanne, wann hab ich mein Examen gemacht, und wann hab ich hier in Österreich angefangen, dann komm ich hier und sie sagen, ich muss nostrifizieren, und muss ich Fächer, die damals auch nicht mal Gegenstand war von der Ausbildung muss ich nachmachen, das hab ich noch verstanden, aber dass zB ein Fach wie Hygiene, ich weiß nicht was die glauben, was in x anders also Hygiene verstanden wird, ob ein Streptokokken anders ist als hier, aber das müsst ich dann nachmachen, und die Gutachterin damals, die Worte, die sie benuztz hat so verletztend, dass sie gesagt hat: Sie brauchen keine Ernährung machen, weil sie ja schließlich 17 Jahre in x gelebt haben. Als wenn wir in x nicht wissen, was eine Vitamine ist und was Ernährung ist, ja. Das sind dann Menschen, die keine Ahnung haben, die wirklich nur auf die Papier schaut, und nicht zwischen die Zeilen auch schauen. Und und wirklich, so (zeigt etwas), ein Pferd sieht noch mehr, weil die haben solche Scheuklappen, dass die nicht mal da sehen mehr was noch dazwischen liegt. 


P10: 
And here you were permanent obviously?
Z	No, I was on an accompanied spouse permit.  So now I had to start looking for a job, I thought it was easy after I had my registration.  I started hopping around every government hospital looking for a job and then I couldn't get anything.

P10: 

What difference does it make?  I mean from your experience, if you've got all the right papers and things, what extra responsibility would they have?
Z	I think the paper work because there is also paper work after they employ you.  Like here, I was employed in October and I started work in April, just this month.

P10: 
Ja I agree.  You just feel lost.  Waiting around. 
Z	You become so desperate that like you asking me do you have a career path?  All I need now is money.  
R	Before you can think about that.
Z	Before I can start thinking, what can I study?  Now I am also pressured to study towards something that would give me more money than something that would satisfy me.  


P18: 

Donc votre diplôme est pas reconnu ici ?
Oui, c'est ça le problème. J'ai essayé de chercher des informations, faire l'équivalence mais le réponse était toujours il fallait refaire la dernière année, ou .. Faire encore des stages.. Tout ce qu'on a fait là, il faut refaire. J'ai dit : c'est pas possible. Et c'est comme ça que j'ai accepté le travail comme aide soignante. 

P23: 

  But the day to day things; my husband's been ill and he needs looking after as all men do and yeah, I'm missing out on so many things now and life is really very lonely here when he's not here.  And there is, yeah, I think, you know, when I look at what I've got, I live in a flat here but when I look at what I've got there and our lifestyle, although that sounds a bit snobbish but it is, it's a different lifestyle, it's an easier lifestyle do you know that, you know, I mean people can work in your home, you know, it's easier to get things done.  Ma


P25: 
When we first came here the….we, it was actually difficult here….as I've said by the time I left x I was a senior nurse so you come to a new country whereby, first of all, people really treated you as an underdog really. I mean I don't like to say but it was the affect that because of your colour; because you came from Africa the third world so they thought that x, we were like the third world.  Somebody would ask me, "Oh where did you buy your clothes, did you buy them at the airport?  Do you wear clothes in x; I thought you wear, you know, those kind of things."  Some nurse would ask me, "Oh," you know I did temperatures, "Oh do you know how to check the temperatures?"  "Yes," "Oh where did you learn how to check temperatures?"  And I was already a trained nurse in here when I came to this country and, "Oh so you know how to check temperatures in x?  The other one they were like the incubator oh "Have you ever touched this incubator" or you know, "Have you seen it before?"  You know those kind of things so god, you know when

P28

P: it was also good; umm it's just that when you are far away from your family, it breaks you sometimes. Because your family is far away, especially when you have children, you don't know how they are what… what… you know.


P28: 


P: hi... hi... (Giggling…) umm anything that I would like to change ha… ha… ha… (Laughing…) work is very far away from the family, sometimes it hurts your mind, if you… if you hear ngwana o a lwala (the child is sick) as a mother you know you would be working thinking oh my child today but if you are near them you know… so if I could have something to change; for those who maybe have got families, maybe to be put where their families are and it depend, they enjoy even working far away with their family.


P29: 
INT: Kalahari-South, ok. Can you tell me the story of your employment after coming to Botswana; umm I know you have mentioned that you worked at the Kalahari, what specialty did you do?

P: the same one, registered mid-wifery.

INT: how was it like there in the Kalahari?

P: the first few days were difficult ha… ha…ha... (Chuckles…) as usual that few days people will not except you…


P29: 
P: totally, you are a… you are a foreigner… he… he… he… (Laughing…) you have come to take the jobs of their children and that was the scenario, some would tell you like that, it was… it wasn't nice. And they would talk to… you know… thinking you are not getting it but I was getting it because my language is similar to tswana so I could catch whatever would be discussed.

P29: 
INT: ok. What about difficulty, was there anything difficult?

P: the only difficult was dis… hmm… distance to where were referring our clients and distance to our home-place.


P30: 

Eventually we coped.  We had difficulties, you know, and those from being, coming from another country and you know as nurses you can't practice if you don't have your practicing license…


P32: 

Yeah, it's beautiful.
R	Yeah.
I	Yes.  But really the thing frustrating by being in foreign lands is being away from your home.
R	Of course.
I	Right.  I'm used to this big comfort zone, my house, but now I'm staying in a small room, you see.

P32
R	Yeah, so, and then how long will it take for you to be naturalized in South Africa.
I	I really don't know.  I think I will take my passport there and ask them if they can give me an ID because as it is now my contract expires on the thirty first of March.
R	Yes.
I	And now when I went to HR they gave me this letter which, it wasn't signed then foreign registration said 'we cannot process the letter because it wasn't signed', I brought it back, it's…
R	Ja, back and forth, back and forth.
I	And my contract is about to expire.
R	Ja.
I	And if it expires, I've got a son, I'm paying five thousand…

P33: 
So when we travelled to South Africa the only challenge we had was our registration with the South African Nursing Council.
R	Okay.
I	It took us almost two years to be registered.

P33
And because we didn't start that process in X, we started it from here but still I didn't expect that while we are within South Africa we should have that long, long…
R	Wait.
I	… waiting because everything was readily available and so there was no justification for that long process.  And so we…

P33
And how did they register you, did they look at your qualifications to give you an equivalent in South Africa or…?
I	Yes, they take our certificates, they go to SAQA and they compare the qualification and then they put us on the right grade with South African standards.  That process went on well but now to get feedback from the nursing council it's hell, it was not easy until we had to travel to and from, to and from and it was costly and also like mentally we were disturbed because we could not do our clinical practice because we needed that registration process first.

P33

and the administration generally?
I	The college, like the institution was somehow supportive but still they were not like, we told them that, at the end of the year we told them that 'there is much more that you can do on behalf of foreign students because South African council and you, you are supposed to be in good terms and so your good communication between each other should facilitate quick rescission…

P33: 
In South Africa maybe it's, sometimes it's the attitude of the nursing staff towards their patients but also vice versa.  And also maybe the attitude of nursing staff versus foreigners.
R	Okay.
I	Yes, that was quite standing out because…
R	Are you talking about foreign patients or foreign nurses?
I	Both, foreign patients and foreign nurses.  Like us, because there were no foreign nurses and it was only, the students, it was, the environment was like because we were like three of us, it was like we made our life comfortable. […]

P37: 
Had you lived anywhere else before here at all?

R:	No.  We'd lived around x.  There was, I had never been out of x so it was a big decision and when we came my daughter couldn't come with us, she had to stay back a year and finish school because she'd; the system's quite different to here so she was, sort of, they go up to first year A-level there and then go straight on to university, and if she came here she'd have to start and do a two years so she decided to stay behind and that was very, very, probably the worst year of our lives with knowing she was there and we were here.

P39: 

Two – working in the ward with somebody who'll tell you that, "Oh you are a registered nurse?"  No but I'd been a carer in this ward for more than ten years so don't come with an attitude of thinking you're going to run this ward.  I'm in charge here, that's the most fascinating thing I ever experienced.  Then where there's an exchange of roles that because I'm a nurse and I qualified in x, together with the fact that I'm black, I cannot work as a registered nurse with the healthcare assistant who's been there for ten years.  I have to do her job then she will do mine.  Hang on who will account here?  It was one of the toughest things but I had to use a positive approach although it was tough because the time I came there were no human rights, there was no act to back my abilities and my understanding of what's acceptable and not except that I would rely on, 'Hey you know there's what is called, the nurse, the governing body for the nurses, if I don't do this, I'll be in trouble'.  Then she'll say, "Even the ones I worked with before, they were trained so don't come with an attitude."  Ah that was the hardest thing in this country so. 

P44: 
We nurses we don't understand sometimes I don't know whether we don't understand or we call it jealous because I faced a lot of problems as am an eye nurse. When I was supposed to be operating as an eye nurse it it…there were some issues which came upon me that I shouldn't practice as an eye nurse. And these issues were just raised from nurses not from the ministry or from the x or what. So there was a lot of compli…complaints and fighting that's why should I work as an eye nurse and then should I just work as a general nurse because I was recruited as a general nurse not an eye nurse…an eye nurse? All these things seriously it was…it was disappointing me because they were  part of the work which I was working, i was trying to save community from enquiring unnecessary blindness which is…we can prevent it but the nurses they didn't focus on that but they just said… it was a lot of talks in fact and it was even…it was a challenge to me and it was even discouraging me saying sometimes I  can go to the matron, I will tell the matron that can I just go to the ward she said no!

P44: 
those kind of countries I mean what should they do in your opinion? Botswana! 
Part: No there is nothing they can do me I can see it's just…it's just to continue with our friendship like we are now yah! Not creating…not to make eh…that here in Botswana they don't like foreigners anymore lets go back to home and we get angry, but we just remain friends yah! But we have to understand that it's a challenge. And we have to understand though we were…we knew that we are not going to die in Botswana, we are going to work at a certain time and at a certain time we need to go back to our country and work there. yah!!

P46
And what in x influenced your decision to move?

R:	For me it was; I thought that the payment is much more than in x but when I came here it was not, it's almost the same.  For you to earn more in UK you have to work hard, long hours, you have to maybe have one, what is it, one day off or you don't; so you have to work hard.  So that is the point that when I thought that the payment was better and also I came because I wanted to study.  That is it, that's what made me to come to this country.

P46
Like in terms of knowing, like here things are expensive.  When we came here everything was easy; getting bank cards, getting what is it, credit cards everything, everything, everything free.  Not now in that these things it's not easy to build up the pound so they must know how to go about through these things.  And also to know exactly and have a time frame; not to engage themselves in all the debts you know.
